# A Review of Hand–Arm Vibration Studies Conducted by US NIOSH since 2000

**DOI:** 10.3390/vibration4020030

**Published:** 2021-06-15

**Authors:** Ren G. Dong, John Z. Wu, Xueyan S. Xu, Daniel E. Welcome, Kristine Krajnak

**Affiliations:** Physical Effects Research Branch, Health Effects Laboratory Division (HELD), National Institute for Occupational Safety and Health (NIOSH), Morgantown, WV 26505, USA;

**Keywords:** hand–arm vibration, hand-transmitted vibration, hand–arm vibration syndrome, vibration biodynamics, vibration health effects

## Abstract

Studies on hand-transmitted vibration exposure, biodynamic responses, and biological effects were conducted by researchers at the Health Effects Laboratory Division (HELD) of the National Institute for Occupational Safety and Health (NIOSH) during the last 20 years. These studies are systematically reviewed in this report, along with the identification of areas where additional research is needed. The majority of the studies cover the following aspects: (i) the methods and techniques for measuring hand-transmitted vibration exposure; (ii) vibration biodynamics of the hand–arm system and the quantification of vibration exposure; (iii) biological effects of hand-transmitted vibration exposure; (iv) measurements of vibration-induced health effects; (iv) quantification of influencing biomechanical effects; and (v) intervention methods and technologies for controlling hand-transmitted vibration exposure. The major findings of the studies are summarized and discussed.

## 1. Introduction

**Figure 1.**
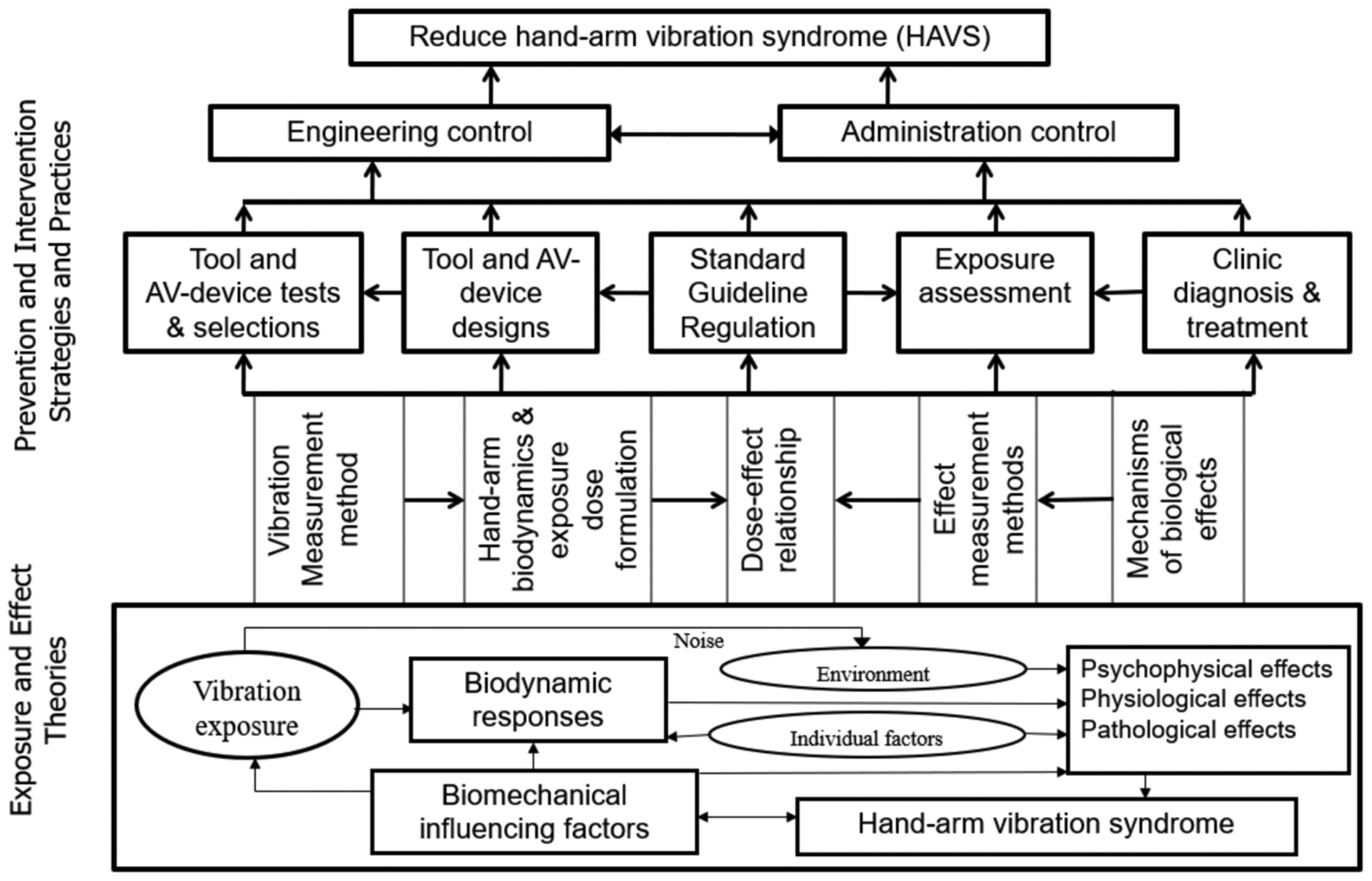
The body of the knowledge on hand-transmitted vibration exposure and health effects.

Prolonged and intensive exposure to hand-transmitted vibration (HTV) is a risk factor for the development of sensorineural, vascular, and musculoskeletal disorders in the hand–arm system, which are collectively called hand–arm vibration syndrome (HAVS) [1,2]; Similar to primary Raynaud’s disease first reported by a French doctor [3], HAVS is typically characterized by tingling and/or numbness followed by cold-induced, painful, episodic finger blanching attacks of one or more fingers, commonly referred to as vibration white finger (VWF) [4]. Symptoms of HAVS were identified in miners using pneumatic vibration tools in Italy [5]. However, the first definitive medical and epidemiological study on HAVS was conducted by Dr. Alice Hamilton in the US [6]. Since then, a comprehensive body of knowledge on HAVS and other musculoskeletal disorders associated with exposure to vibration has been reported, as shown in Figure 1. However, there are still many questions remaining regarding the etiology of the disorder, and how the specific components of vibration exposure contribute to the risk of developing HAVS.

In the US, the National Institute for Occupational Safety and Health (NIOSH) has contributed significantly to understanding the risks of working with vibrating hand tools and handheld vibrating workpieces, publishing a number of studies examining hand-transmitted vibration (HTV). While a few were studies performed by researchers in different Divisions of NIOSH [7,8], most of NIOSH’s intramural projects have been carried out in two systematic research programs. The first program was conducted by a research team led by Don Wasserman in Cincinnati, OH, from 1972 to 1984 [9]. This program emphasized the epidemiological study of HAVS. The results of this research confirmed that HAVS remained one of the major occupational diseases among workers exposed to HTV in the US [10,11]. These studies formed the basis of the NIOSH criteria and recommendations regarding occupational exposure to hand–arm vibration [12,13]. This research program also contributed to the establishment of original national and international standards and guidelines on the measurement and assessment of HTV exposure [14–16]. The second research program that began in 2000 is ongoing and performing studies characterizing the vibration and how it influences the development and severity of HAVS. This research program has been conducted by researchers in the Health Effects Laboratory Division (HELD). Different from the first program, the second program has emphasized fundamental biodynamic and biological research along with engineering intervention studies. While the detailed information from the first NIOSH research program can be found in the review by Wasserman and Reynolds [9], the current review focuses primarily on the studies performed in the 20 years of the second NIOSH HTV research program. This review describes the general concepts and hypotheses of the reviewed studies, summarizes and discusses the findings in relation to current knowledge about the relationship between biodynamics and the health effects of HTV, and identifies research gaps.

## 2. General Concepts and Hypotheses

It has been well established that vibrating an engineered structure (e.g., bridge frames, car axles, and airplane wings) may result in fatigue damage. The fatigue life of the structure depends on the fatigue resistance of the structural material and the vibration exposure dose that can be formulated primarily based on the vibration stress (the vibration force per unit area of structural material) and/or strain (the vibration deformation per unit length of the structural material) at critical locations inside or on the structure, the number of the stress cycles, and the quasi-static stresses and strains at the critical locations [17]. Because the human hand–arm system is also susceptible to fatigue induced by physical stressors, we hypothesized that the development of HAVS may also be conceptually considered as a long-term fatigue process [18]. This hypothesis is consistent with the fatigue-failure theory to musculoskeletal disorders proposed by Gallagher and Schall [19].

As illustrated at the bottom (Exposure and Effect Theories) of Figure 1, the onset of any vibration effect in the hand–arm system generally includes two sequential processes [1,18]: (i) biodynamic responses (stresses and strains) to the vibration input into the hand; and (ii) the health effects that are a result of those biodynamic responses. Because the vibration responses of the hand–arm system are similar to those of engineered materials, the methods used for calculating the vibration responses and exposure dose are similar for both types of structures. The vibration stresses or strains are superimposed on the quasi-static stresses or strains induced by applied hand or body forces. Like any engineered material, the tissues of the hand–arm system also may be injured or display maladaptive changes in physiological function when the combined stresses and/or strains are beyond certain levels. Unlike engineered materials, the human body can repair the injuries and adapt to vibration exposure through a series of complex biological responses; long-term disorders or symptoms of the HAVS occur when the living tissues cannot repair the injuries and/or restore normal function. Additionally different from engineered materials, the human nervous system can transmit vibration information from regions of the body that are exposed to vibration to the brain and other regions that are not directly exposed through changes in the activity of the sympathetic nervous system [20,21]; the injury or malfunction of a blood vessel at one location may also affect the blood circulation at other locations. These differences do not substantially change biodynamic responses of the hand–arm system to vibration, or the basic formulation of the vibration exposure dose, but these biological factors make the mechanisms underlying the development of HAVS much different and more complex than the mechanisms underlying the fatigue of engineered materials. As also illustrated in Figure 1, besides the vibration exposure factors (vibration magnitude, frequency, direction, and exposure duration), the biological responses to vibration may also be influenced by environmental factors (temperature, noise, and moisture), biomechanical factors (hand coupling forces, hand contact pressure distribution, and hand and arm postures), and individual factors (genetics, tobacco use, age, sex, hand and arm injury history, and individual biodynamic properties) [1,2]. As a result, the development of HAVS is much more complex than the fatigue response of any engineered structure.

More critically, while the fatigue life of an engineered material can be determined through laboratory fatigue experiments, it is not ethical to induce HAVS in human subjects in laboratory experiments. It would also be difficult to replicate all the factors that contribute to the development of HAVS in the laboratory. Therefore, other approaches must be considered when characterizing dose–effect relationships of HAVS and determining how those relationships contribute to the risk of developing vibration-induced disorders. Epidemiology studies were one of the approaches primarily used in the first NIOSH research program to describe the relationships between vibration and the development of HAVS [10,11]. In these studies, the vibration input to the hand and influencing factors (the exposure factors included in the ellipses in Figure 1) were measured or estimated, together with the survey and/or examination of health outcomes or hand–arm vibration syndrome among workers exposed to HTV. The data were used to assess the relationships among the vibration exposures and outcomes. Although many epidemiological studies have established the qualitative association between hand-transmitted vibration exposure and HAVS [7], they have not established a reliable quantitative dose–effect relationship for any component of the disease [22]. As stated in the current ISO standard [4], the vibration exposure dose needed to induce disorders associated with HAVS is not precisely known, neither with respect to vibration magnitude, frequency spectrum, and direction, nor with respect to daily or cumulative exposure durations. This may be in part because vibration exposure is often accompanied by other exposures in the workplace, and it is difficult to determine the contribution of these varying factors to the development of HAVS when developing an exposure formula. Other possible reasons that epidemiological data may not accurately describe the dose–response relationship between vibration and injury include: consistent methods may not have been used to measure vibration exposure factors; the exposure dose formulas used in these studies may not have accurately reflected the true biodynamic responses of the exposed tissues; and the methods used for identifying or quantifying HAVS symptoms may not be reliable.

These observations suggest that it is important to enhance the understanding of HTV and its relationship to various health effects by developing more reliable methods and techniques for measuring vibration exposure, health effects, and their influencing factors, and to improve the formula for quantifying the exposure and the vibration assessment method. This can be achieved by systematically studying the biodynamic and biological processes. Therefore, the NIOSH HTV research program includes one group focused primarily on characterizing the biodynamic effects of vibration, while the other is focused on determining how the biodynamic effects may be related to the biological effects of vibration.

## 3. The Standard Method for Measuring and Assessing Hand-Transmitted Vibration Exposure

The vibration transmitted to a hand can be characterized using four factors: vibration magnitude, exposure duration, vibration frequency, and vibration direction. The standard risk-assessment method requires measuring vibration acceleration in root-mean-square values on a tool or workpiece in the hand contact area in three orthogonal directions. Their vector sum is used to represent the vibration magnitude. It is weighted using a frequency weighting function defined in the standard to yield frequency-weighted acceleration (A_w_). The standard method also requires quantifying the daily vibration exposure duration. It, together with the weighted acceleration value, is used to calculate the daily exposure dose or A(8) value. In many countries, the A(8) value is required to be less than 5 m/s^2^ (Limit Value); vibration-reducing interventions are also required if the A(8) value is greater than 2.5 m/s^2^ (Action Value) [23,24].

The development of a convenient and reliable method for measuring the vibration exposure duration remains an important research task. Self-reported data could largely overestimate the actual exposure duration [25,26], which could be one of the major sources of error in some reported exposure doses. A convenient and objective method is desired to measure the exposure duration. A vibration wristwatch may be used to accurately measure the actual exposure time [27]. It may also serve as a direct-reading device or a vibration dosimeter that can be used to help monitor and control vibration exposures. Studies have been performed to establish a theoretical basis and to examine accelerometer mounting techniques on the hand [28,29]; these studies are presented in Section 4, along with other methods for quantifying vibration exposure based on the biodynamic response.

Another major source of errors in reported vibration data is the baseline drift or dc-shift of the acceleration signal measured using piezoelectric accelerometers, especially on impulsive tools [30,31]. The dc-shift may cause an overestimation of the vibration magnitude, especially frequency-weighted acceleration, because the current frequency weighting function emphasizes the vibration components in the low-frequency range (≤25 Hz) [4]. This was confirmed in a study comparing the vibration spectra measured with a conventional accelerometer with those measured using a laser vibrometer [32]. The study found that it was difficult to sufficiently control the dc-shift using available commercial mechanical filters. Instead, insertion of a layer of rubber between the accelerometer and the tool handle and adjusting the accelerometer mounting tightness until the low-frequency component at 5 Hz or 6.3 Hz in the one-third octave bands is below an acceptable value (e.g., <1.0 m/s^2^ for the data measured on a chipping hammer handle with a fundamental vibration frequency at 25 Hz or higher). Increasing the thickness of the rubber and/or reducing the mounting tightness can further reduce the low-frequency error but it may substantially increase measurement errors at high frequencies (>500 Hz).

**Figure 2.**
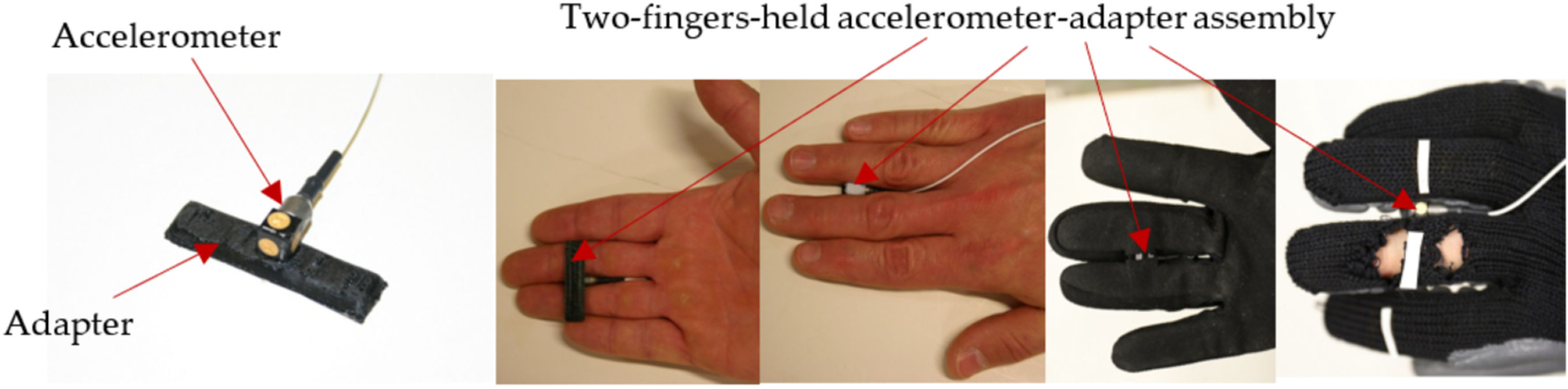
A two-fingers-held adapter for measuring the vibration input to the hand [35].

The most convenient approach for measuring vibration on a tool handle or handheld workpiece is to use a finger- or palm-held adapter equipped with a tri-axial accelerometer, especially when a glove is used to position the adapter. The typically available adapters, including some of those recommended in ISO 5349-2 [33], were evaluated [34]. The results suggest that many of the handheld adapters may produce major overestimations of vibration exposure, especially in the most important middle frequency range (16 Hz to 200 Hz). These measurement errors may significantly vary with tool, adapter model, mounting position, mounting orientation, and subject. The primary problems with this approach include the unavoidable influence of the dynamic motion of the hand on the adapter, unstable attachment, insufficient attachment contact force, and inappropriate adapter structure. However, the results of this study also suggest that measurement errors can be reduced if the design and use of an adapter are systematically optimized toward minimizing the combined effects of the identified factors. The proposed requirements for the optimized design of the adapter are as follows: (i) the mass of the adapter and its tri-axial accelerometer should be as small as possible; (ii) the profile of the adapter should be as low as possible, and the accelerometer should be installed on the adapter as close to the contact surface as possible; (iii) the adapter configuration should allow for a sufficient force to be applied on the adapter to prevent separation of the adapter from its contact surfaces under vibration; (iv) the adapter should not change the original hand postures; (v) the vibration transmissibility measured with the accelerometer fixed on the adapter without coupling with the hand should be close to unity in the entire frequency range of concern (5 Hz to 1500 Hz) with a maximum error at <5%. Guided by these requirements, an adapter with a tri-axial accelerometer located between two fingers was developed [35], which is shown in Figure 2. It has been successfully used to test and evaluate the vibration transmissibility of vibration-reducing gloves at the fingers. The results suggest that the adapter on an ordinary work glove remained at near unity transmissibility in the entire frequency range of concern. This suggests that such an adapter can be built into a glove to conduct convenient and efficient vibration measurements, especially if the instrumented glove can be equipped with a wireless device for data transmission to a smart phone, similar to a commercially-available instrumented glove for vibration measurement [34]. The accelerometer shown in Figure 2 should be replaced with a more robust accelerometer if used to measure high vibration magnitudes or shocks on percussive or impulsive tools.

**Figure 3.**
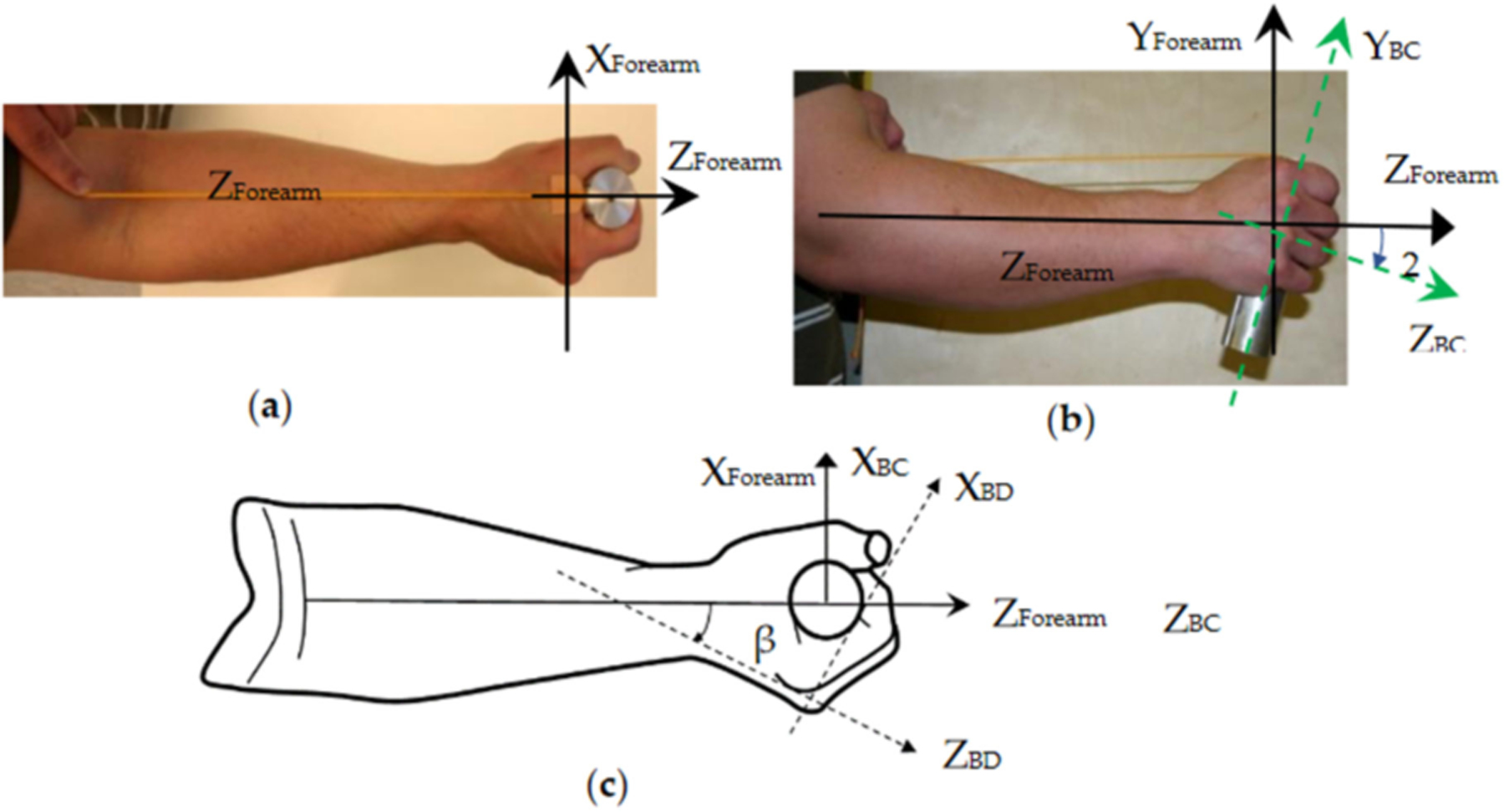
Comparisons of the proposed coordinate system (Forearm) with the standard hand coordinate systems (BC and BD) [39]: (**a**) The definition of the forearm-based coordinate system; (**b**) The comparison of the coordinate systems in the Y-Z plan; and (**c**) the comparison of the coordinate systems in the X-Z plan.

Although the current standard risk-assessment method requires the measurement of the vibrations in three orthogonal directions, the vibration direction has not been considered in the risk assessment, as the vibration accelerations in the three directions are considered equally important in the calculation of the exposure dose [4]. The biodynamic responses of the hand–arm are direction-specific [36,37]; this suggests that the vibration direction may also contribute to the development of vibration-induced health effects and should be considered in future studies. This requires improvement of the hand coordination systems defined in the current standards. Two types of coordinate systems have been defined and used in human vibration studies [4,38]: (I) a basicentric (BC) coordinate system is defined on the equipment or tool primarily for guiding the installation of an accelerometer on its human body or hand contact surface to measure the vibration input to the human body or segments; and (II) an anatomically based biodynamic (BD) coordinate system is defined primarily for describing, measuring, and analyzing the body or segment postures and its biodynamic responses. The hand coordinate systems defined in the current standards were systematically reviewed and evaluated, which produced the following findings and recommendations [39]: (i) the standard BC coordinate system is defined by using the tool action direction as the first reference and the handle axial direction as the second reference; the reference sequence should be changed or the handle axial direction should be considered as the primary reference because the accelerometer is usually installed on the tool handle; (ii) different from whole-body vibration exposure in which the BD coordinate system is usually naturally aligned with the BC coordinate system [40], the hand BD coordinate system has a different orientation from the tool-based hand BC coordinate system; such difference may also vary with tool types, models, tasks, and operating conditions [39]; hence, it is inconvenient and difficult to use this hand BD coordinate system as the primary coordinate reference in HTV studies; this explains why the standard hand BD coordinate system has been rarely used in practice; (iii) to minimize the difficulty, a forearm-based BD coordinate system is defined [39], as shown in Figure 3a,b. Such a coordinate system can be used as the primary coordinate reference to control the vibration exposure direction in a laboratory experiment, to measure the hand forces, and to help estimate the hand and arm postures in a tool operation. In fact, the proposed coordinate system has been used in many biodynamic studies and in several standards [41–43], because it is easily visually identifiable and practically implementable. For example, the forearm axial direction is required to be aligned with the vibration direction on a single-axis test system in the standard anti-vibration glove test [42]. This is equivalent to requiring the forearm-based BD coordinate system to be aligned with the hand BC coordinate system, as shown in Figure 3c. To achieve the alignment, the hand must rotate about 20° in the Y_Forearm_-Z_Forearm_ plane from its neutral posture. As also shown in Figure 3c, there is an obvious angular relationship (β: ≈ 30° for a 40 mm cylindrical handle) between the standard hand BD coordinate system and the forearm-based BD coordinate system [39]. These hand–arm postures and the forearm BD coordinate system have also been used in the measurements of glove vibration transmissibility and biodynamic response functions on a 3D vibration test system [36,37,44]. We recommend the use of these postures and this coordinate system to replace the standard hand coordinate systems or consider them as alternative hand–arm coordinate systems in further revisions of the standards.

## 4. Vibration Biodynamics of the Hand–Arm System and Alternative Methods for Quantifying Vibration Exposure

The goals of hand–arm vibration biodynamic research are: (I) to measure the biodynamic response functions and to identify the biodynamic properties of the hand–arm system that can be used in the design and analysis of powered hand tools and vibration-reducing devices; (II) to provide data and information that can contribute to the formulation of the vibration exposure dose based on the biodynamic theory; and (III) to help understand the vibration effects.

Ideally, the detailed biodynamic responses such as vibration stress, strain, and power absorption density (VPAD) of the hand–arm system should be measured and used as a basis to quantify the vibration exposure dose. However, it is extremely difficult to directly measure these responses. Alternatively, they can be predicted using biodynamic response functions such as apparent mass, mechanical impedance, and vibration transmissibility of the hand–arm system. These response functions can be directly measured in an experiment or estimated by using a computer model of the hand–arm system that is calibrated or validated using the directly measurable response functions of the system. Hence, the first essential task of biodynamic research is to measure these response functions. These advanced measurement methods are described in Section 4.1. A finite element (FE) model of the hand–arm system or its substructures can be used to predict the detailed distributions (vibration stress, strain, and VPAD). The reviews of the FE model developments and applications are presented in Section 4.4. As FE modeling is technically demanding, expensive, and time-consuming, the development of an FE model for the entire hand or hand–arm system remains a formidable research task. As an approximate but efficient approach, the biodynamic responses distributed in the system can be estimated using a lumped-parameter model of the system. A review of the lumped-parameter model developments is presented in Section 4.2. A review of the alternative vibration exposure measures using the lumped-parameter model and/or directly measurable vibration responses are presented in Section 4.3. The frequency response functions represent the overall biodynamic properties of the system; hence, the measured response functions and/or models calibrated using these functions can be used to help design and analyze powered hand tools and vibration-reducing devices. While the developments of these related models are reviewed in Section 4.2, their applications for interventions are described in Section 6.

The ratio of a biodynamic response (BR) and the vibration acceleration (A_Tool_) input to the hand is conventionally called the transfer function (Tr), which usually varies with vibration frequency. In other words, their relationship can be generally written as follows: BR = Tr·A_Tool_. This transfer function is effectively the frequency weighting of the biodynamic response; hence, it is termed as a biodynamic frequency weighting [45]. For example, the vibration transmissibility measured at the wrist can be considered as the biodynamic frequency weighting of the wrist vibration acceleration. Because many vibration effects are the result of biodynamic responses, the frequency dependency of the biodynamic process is an essential part of the frequency dependency of the vibration effect [18]. For example, the vibration perception on the forearm generally decreases with the increase in the frequency under a constant-acceleration vibration exposure. This is primarily because the forearm vibration transmissibility generally decreases with the increase in frequency [28,37]. If the biodynamic frequency weighting is reliably identified from laboratory studies, it can be used to estimate the BR or quantify the vibration exposure from the vibration acceleration measured on a tool at a workplace, like the calculation of the frequency-weighted acceleration required in the standard risk-assessment method [4]. Therefore, biodynamic research can help to create alternative frequency weightings for assessing the risk of HTV exposures. Although the standard frequency weighting was not established based on the biodynamic concept, it is likely to include the biodynamic frequency weighting. This is explained and discussed in Section 4.3, together with the reviews of other biodynamic frequency weightings.

### 4.1. The Measurement of the Biodynamic Responses of the Hand–Arm System

**Figure 4.**
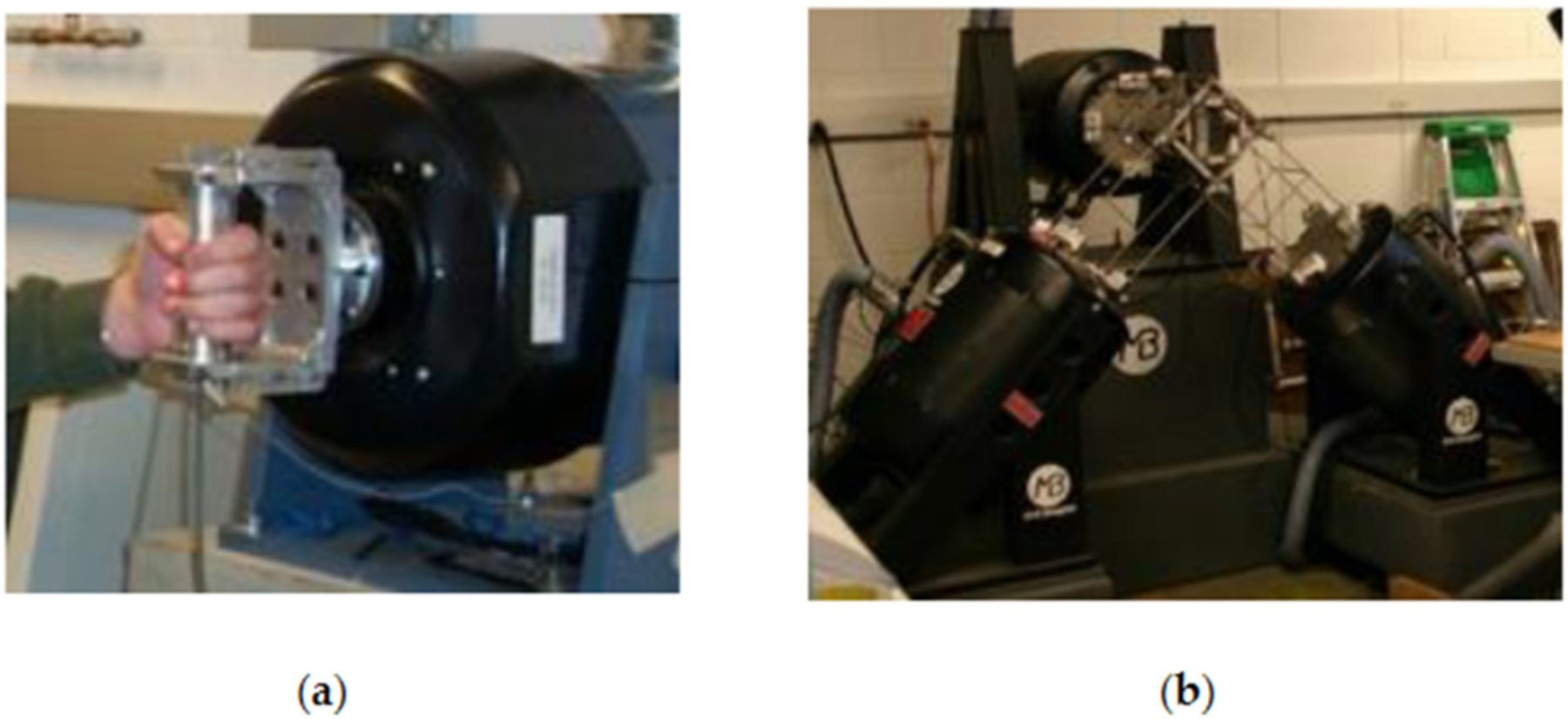
The hand–arm vibration test systems in NIOSH: (**a**) 1D system; and (**b**) 3D system [46].

As shown in Figure 4, the human hand–arm vibration laboratory in NIOSH is equipped with two 1D hand–arm vibration test systems and a unique 3D hand–arm vibration test system. NIOSH researchers initiated the development of the 3D system [46]. It has been successfully used in many experiments.

**Figure 5.**
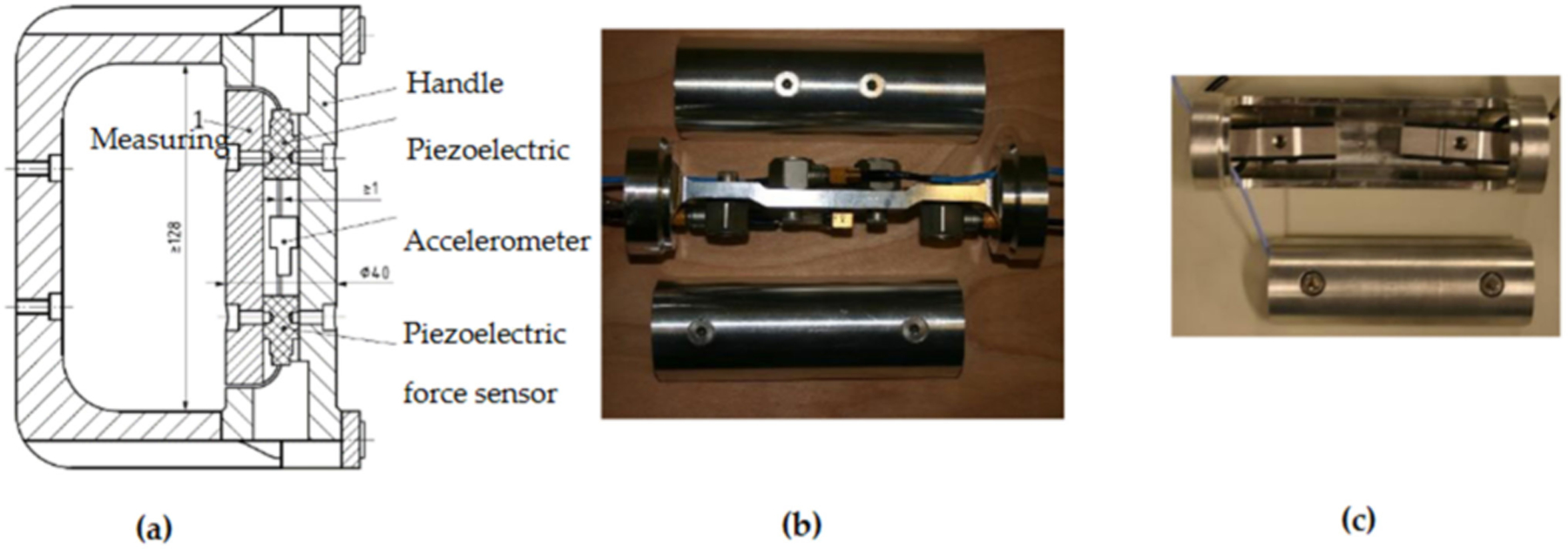
Examples of the instrumented handles developed and used by NIOSH researchers [52]: (**a**) The instrumented handle with the highest resonant frequency; (**b**) The instrumented handle for simultaneously measuring the responses at the fingers & palm of the hand; and (**c**) The instrumented handle equipped with strain-gauge force sensors.

Vibration-induced neurological and vascular disorders in the fingers are major components of HAVS [1,2]; this indicates that the biodynamic and biological responses of the fingers are different from other parts of the hand–arm system. Hence, it is essential to quantify the finger vibration exposure by measuring and/or modeling the finger biodynamic response functions. Unfortunately, little attention was paid to this critical feature before NIOSH’s study of hand vibration biodynamics [47]; only the driving point response functions of the entire hand–arm system were measured and used to develop previous models of the hand–arm system [48]. Furthermore, some of the biodynamic response functions reported in the literature are questionable [49]. Without reliable experimental data, it is impossible to develop an accurate model of the hand–arm system. Upon recognizing these deficiencies, NIOSH researchers developed a novel instrumented handle to accurately measure the apparent masses or mechanical impedances distributed at the fingers and palm of the hand [47,50], which is shown in Figure 5a. The research examined handle dynamics and demonstrated that the sum of the distributed impedance is equal to the impedance of the entire hand–arm system [51]. It also demonstrated that the accuracy of the biodynamic measurement in the high-frequency range depended on the rigidity of the handle or the fundamental natural frequency of the handle. Because this handle’s natural frequency is above 1750 Hz [52], it provides accurate measurement in the entire frequency range of the concern (5 to 1500 Hz) for standard risk assessment of HTV exposures [51]. The design of this handle has also been adopted in the standard anti-vibration glove screening test [42]. To increase the measurement efficiency, a second instrumented handle was developed to simultaneously measure the driving point response functions distributed at both the fingers and the palm of the hand [53], as shown in Figure 5b. This handle is acceptable for the measurement of the response functions at frequencies ≤ 1000 Hz. A third instrumented handle was specifically designed for the 3D test system for measuring the driving-point response functions in the three orthogonal directions [36,52].

To assure the rigidity of the instrumented handles, each handle is equipped with piezoelectric force sensors. Their baseline signals are sensitive to changes in the handle temperature, which may be induced from the difference between the handle temperature and hand temperature. This issue has been minimized by resetting the baseline of the grip force measurement to zero before each measurement trial [50]. This method can largely reduce the temperature-induced measurement errors during short testing durations (<1 min). To eliminate the temperature issue in the experiment that requires a subject to continuously hold the handle for a long duration (e.g., >3 min), another instrumented handle equipped with strain-gauge force sensors has also been developed and used in some of the experimental studies [54–57]. The strain-gauge handle is useful for experiments concerning vibration exposure frequencies of ≤750 Hz. The push force is usually measured using a force plate. The grip and push forces are typically processed and displayed using an in-house developed LabVIEW program.

While it is difficult to measure the vibration inside the hand–arm system or in the skeletal portion of the system, the vibration on the skin of the system can be directly measured. These skin surface measurements can be used as an alternative measure for quantifying the vibration exposure [58], and to calculate the vibration transmissibility of the hand–arm system [59,60]. This on-the-hand method can also be used to evaluate the effectiveness of vibration-reducing devices [61]. A 3D scanning laser vibrometer has been used to measure the vibrations on several skin surface locations along the hand–arm system [37,54,62]. Alternatively, the vibrations in the three orthogonal directions can be measured using a tri-axial miniature accelerometer attached to the skin on the hand–arm system. These accelerometers are much less expensive and more convenient and applicable to the experiments both in a laboratory and at a workplace; however, a major concern is that the accelerometer and its attachment device may affect the test results. To address this issue, the accelerometer method was examined in an experimental study [29]. In the experiment, adapters equipped with accelerometers were attached to the wrist, forearm, and upper arm for the measurements. The measured data were compared with those measured with a laser vibrometer without the adapter attachments. The results suggest that the two technologies provide comparable results with similar basic trends and the differences due to adapter mass and/or fastening tightness can be minimized [29].

With the above-described methods and technologies, experiments have been conducted to measure the driving point response functions distributed at the fingers and palm of the hand [36,47,63–66], and/or the vibration transmissibility spectra at several locations on the hand–arm system [28,37,54,62,67–69]. The effects of influencing factors such as hand forces, hand–arm postures, and vibration direction on the response functions were also examined in some of these experimental studies. The volumes and sizes of the fingers, hand, and forearm were also measured in the experiments, which made it possible to estimate the average VPAD and hand vibration contact stress and strain.

### 4.2. The Development of Lumped-Parameter Models of the Hand–Arm System

One of the problems with mechanical equivalent models of the hand–arm system reported before 2007 was that the fingers were not considered as a separate element in the models [48]. This made it impossible to determine the unique characteristics of the finger vibration responses in the simulations. Because these models may approximately simulate the measured apparent mass or mechanical impedance of the entire hand–arm system, they are classified as mechanical-equivalent models. They may be used to improve tool designs and analyses [41,70], as only the overall apparent mass or impedance is of concern in such cases. However, these models may not provide reasonable predictions of the vibration responses distributed in different substructures of the hand–arm system. This issue has been addressed through the development of the novel lumped-parameter model of the hand–arm system [71]. A novel theorem of the relationship among driving point response functions and vibration transfer functions distributed in the human body has also been established, which significantly enhanced the vibration modeling theory [72]. This modeling methodology has been improved based on the modeling theory and a set of criteria for calibrating and validating human vibration models using the measured frequency response functions of the system has been proposed [73].

**Figure 6.**
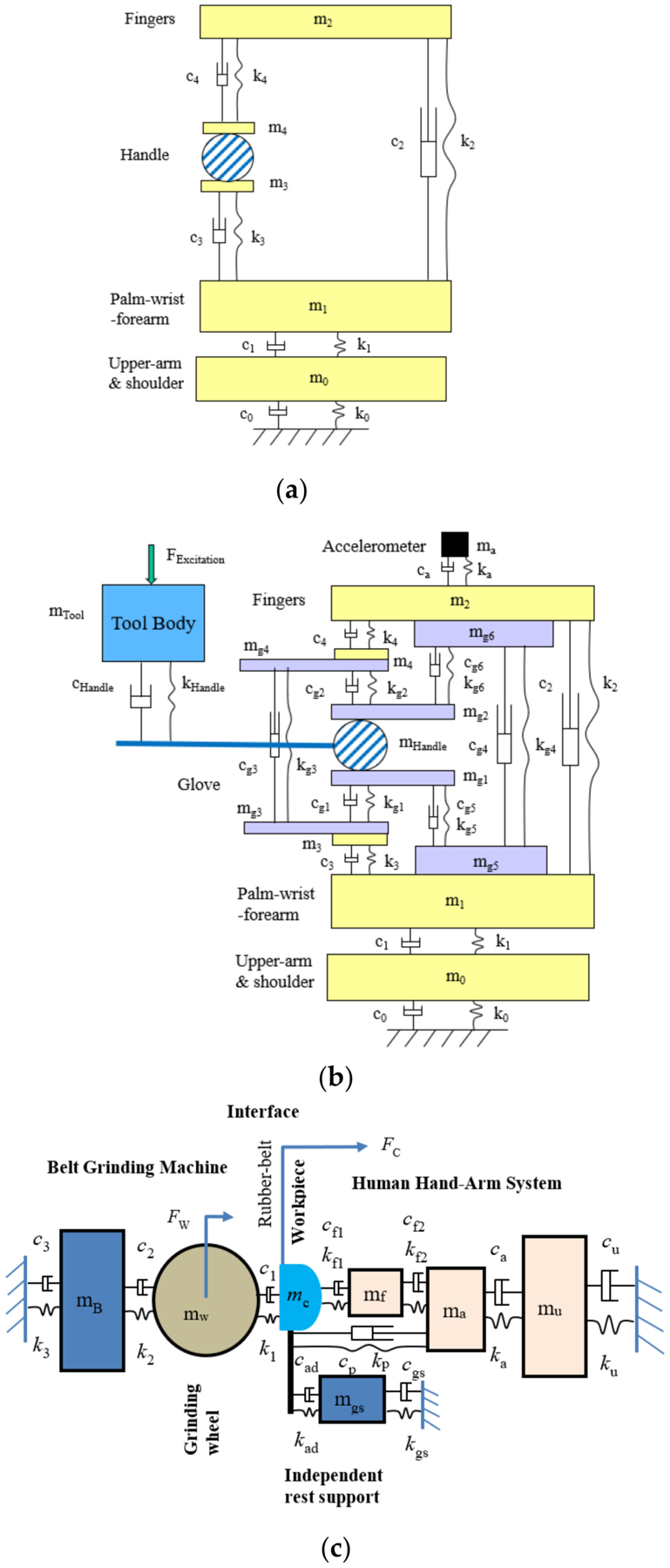
The lumped-parameter models of the hand–arm system developed by NIOSH researchers: (**a**) a model of the hand–arm system [71]; (**b**) a model of the entire tool–handle–glove–hand–arm system [77].; and (**c**) a model of grinding-machine–workpiece–hand–arm system [78,79].

Figure 6a shows an example of the novel lumped-parameter models of the hand–arm system [71]. This model is applicable for simulating the vibration responses of the hand–arm system in each of the three orthogonal directions [74]. This model has been used to study the vibration power absorption distributed in the major substructures of the hand–arm system and to derive the biodynamic frequency weighting described in the next section [75]. This model has also been used to evaluate published experimental data, select specific data sets, and synthesize the data for updating ISO 10.068 [49,76]. The synthesized data in each vibration direction, together with their corresponding models, have been included in the updated standard on mechanical impedance of the hand–arm system [41]. This model has also been extended to include a glove model to study the mechanisms of vibration-reducing gloves [53]. An updated version of the model for the entire tool–glove–hand–arm system is shown in Figure 6b [77]. The hand–arm system model has also been included in the model of the entire grinding-machine–workpiece–hand–arm system for simulating handheld workpiece vibration and for identifying and analyzing effective intervention methods [78,79], which is shown in Figure 6c. However, the complex model shown in Figure 6a is not needed for the construction of a physical hand–arm simulator for tool vibration testing; rather a simple mechanical-equivalent model is well suited for this purpose [80]. This is because the model for the design of the physical simulator should be as simple as possible not only because it is difficult and expensive to build a comprehensive physical simulator of the system but also because it is not necessary to do so. The effective mass of the hand–arm system is usually less than 200 g at frequencies above 200 Hz [36,41,49]; inaccurate simulations resulting from the simple model are unlikely to have a substantial impact on the vibration behaviors of many tools, especially large tools.

### 4.3. Alternative Measures of Vibration Exposure and Their Biodynamic Frequency Weightings

#### 4.3.1. Vibration Acceleration on Hand and Arm Substructures

The vibration acceleration that can be directly measured on the skin at a location on a substructure (A_S_) of the hand–arm system is the simplest biodynamic response that can be directly used to quantify the vibration exposure dose [58,81]. It may be generally termed as on-the-hand–arm-system method. This method assumes that vibration acceleration measured on the surface of the system may be approximately representative of the overall biodynamic responses distributed in the tissues in some areas of the hand–arm system. As mentioned above, the vibration transmissibility is the biodynamic frequency weighting of this vibration measure (W_TR_) [45]. There are two approaches for implementing this method: (i) to predict the acceleration using the measured vibration transmissibility and the tool/workpiece vibration acceleration (A_Tool_) or A_S_ = W_TR_·A_Tool_; and (ii) to directly measure the vibration acceleration using a miniature tri-axial accelerometer attached to the skin surface at a measurement location on the hand–arm system. The first approach is like the standard method for calculating the weighted acceleration, except that its weighting (W_TR_) is generally different from the standard hand–arm frequency weighting (W_h_). The reported vibration transmissibility spectra have made it possible to determine the W_TR_ for each major substructure of the hand–arm system for crudely estimating the substructure-specific vibration exposure dose. The substructure weightings may be used to explore relationships between the exposure dose for the substructure and the substructure-specific health effects.

The second approach not only automatically considers some influencing factors such as hand forces and hand–arm postures but also avoids other measurement issues such as the dc-shift and the hand interface interference associated with the tool/workpiece vibration measurement. This approach can also be more efficient than the standard measurement method, especially when a wearable vibration dosimeter is used to conduct the measurement [82]. Such a vibration dosimeter can also be used as a monitor for controlling HTV exposures. However, besides the above-mentioned uncertainties of the vibration measurement on the hand–arm system, this approach also has a fundamental limitation: the vibration acceleration measured at limited locations on the system may not be sufficiently representative of the biodynamic responses at every location in the hand–arm system. While it is very difficult to fully resolve this issue, the proposed first solution is to measure the vibration acceleration on the wrist if the measurement can only be conducted at one location. This measurement location is desirable for two reasons: (a) the vibration measurement at the wrist has minimal interference with most tool operations; and (b) the accelerations measured at the wrist when operating a number of different tools are likely to be correlated with the standard frequency-weighted accelerations because the wrist vibration transmissibility exhibits some similarities to the standard frequency weighting [28,29,37]. For these reasons, the on-the-wrist method may be acceptable for a preliminary or crude risk assessment of HTV exposures. It may also be a feasible method for the long-term monitoring and control of HTV exposures. Published studies have been used to develop the on-the-wrist method and a related standard [82]; some wrist vibration measurement devices have been available. It should also be noted that the on-the-wrist method has the same major deficiency as the standard weighted acceleration method: neither method provides an acceptable measure of the finger vibration exposure. The finger vibration acceleration should be measured for assessing the risk of finger vibration disorders when the technologies are further advanced to develop a sufficiently small, reliable, and affordable finger vibration dosimeter. This knowledge and information may be used to develop an international standard on the on-the-system method.

#### 4.3.2. Vibration Force and Average Vibration Stress

The vibration biodynamic forces (F_D_) distributed on the finger and palm contact areas can be estimated from the measured apparent mass (AM) and the vibration acceleration (A_Tool_) input to the hand or F_D_ = AM·A_Tool_ [83]. The apparent mass on each contact area is the biodynamic frequency weighting of the contact vibration force on the contact area or W_F_ = AM. At the palm side, the apparent mass has its major resonance in the frequency range of 10 to 50 Hz [36,63,66]. Many percussive tools such as rock drills, road breakers, and chipping hammers generate their dominant vibrations in this frequency range. Therefore, the vibration force acting on such tools can be substantial or comparable with the applied hand forces [83]. This explains why the hand and arm may feel heavy when operating such tools. More importantly, the large magnitude of the combined force (applied force + dynamic force) may cause injuries to the hand–arm system.

The average contact vibration stress or pressure (σ_Av_) at the fingers or palm of the hand can be estimated using the vibration force and the finger or palm contact area (A_Con_) or σ_Av_ = F_D_/A_Con_. The vibration exposure dose rate (V_Dose-rate_) can be taken as the stress rate at each frequency, which can be calculated using the stress and the frequency value (*f* = the number of cycles per second) or V_Dose-rate_ = σ_Av_·*f* = (2π*f*·AM)·[A_Tool_]/[2πA_Con_]. It may be used as a vibration measure to quantify the vibration exposure in the finger and palm contact soft tissues [84]. Naturally, the mechanical impedance (MI = 2π*f*·AM) is representative of the biodynamic frequency weighting of this exposure measure or W_Stress-Rate_ can be determined by normalizing MI.

#### 4.3.3. Total Vibration Power Absorption

**Figure 7.**
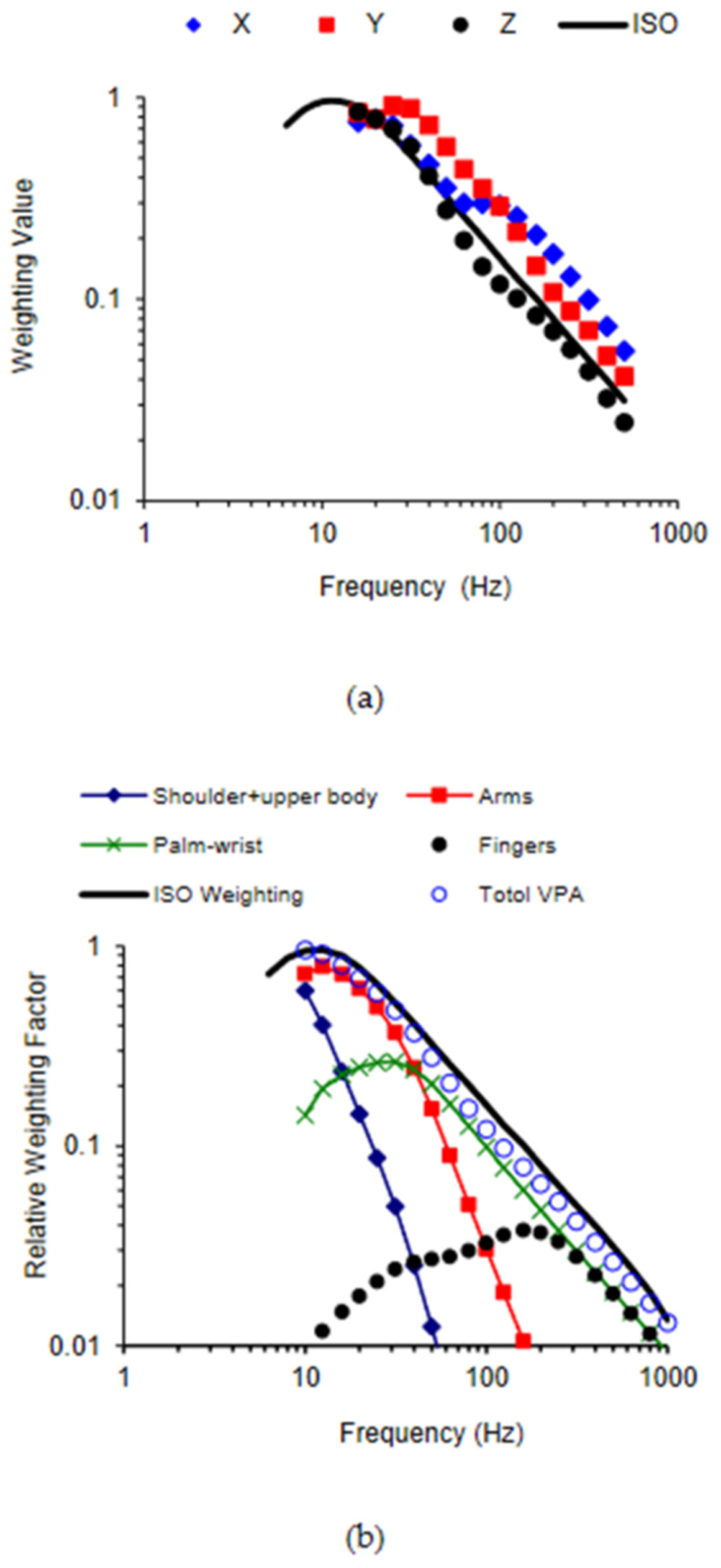
Comparisons of the VPA and standard frequency weightings: (**a**) the total VPA weightings in three orthogonal directions (X, Y, and Z in the forearm-based hand coordinate system shown in Figure 3) [36]; and (**b**) the relationships among the total VPA and the VPAs in the major substructures of the hand–arm system along the forearm direction (*z*-axis) [75].

The total vibration power absorption (VPA) method was initially proposed in the 1970s [85,86]. The VPA of the entire hand–arm system can be quantified using two approaches [86–89]: (i) directly measured using an instrumented handle; and (ii) estimated using the real part of the measured mechanical impedance and the vibration acceleration input to the hand. However, some questions regarding this method were raised after our lab observed substantial differences between the finger VPA and the total VPA in our experiments [47,64]. Our studies also demonstrated that the total VPA is similar to the standard frequency-weighted acceleration because their frequency weighting functions are similar to each other [36,90,91], as shown in Figure 7a. The major advantage of the total energy method is that the biomechanical factors such as hand forces and hand–arm postures, which may vary during tool operation, can be automatically considered in the VPA data if they are directly measured at workplaces. However, it is much more difficult to measure the VPA than to measure the vibration acceleration because an instrumented handle is required to measure both dynamic force and acceleration during tool operation.

The similarity between the curves shown in Figure 7a contributed to our understanding of the standard frequency weighting and its appropriate applications. The original definition of the standard frequency weighting was largely influenced by the frequency dependency of the equivalent sensation and comfort contours reported by Miwa [92–95]. Later, it was slightly modified to its current shape. Specifically, it has an approximately constant acceleration from 8 Hz to 16 Hz and a constant velocity from 16 Hz to 1000 Hz. The constant velocity hypothesis in the major frequency range of concern was supported by the results of some other studies on the vibration sensation and comfort of the entire hand–arm system [96–98], as the equivalent sensation or comfort of the system approximated towards a constant velocity when the vibration velocity was above a certain level. Therefore, the standard frequency weighting is approximately a frequency weighting of the vibration discomfort or pain of the entire hand–arm system. Its strong similarity to the frequency weighting of the total VPA suggests that the biodynamic responses play an essential role in determining the vibration discomfort or pain. This supports the above-described general concept and hypothesis of HTV exposure and vibration effects.

Figure 7b shows the relative VPA distribution frequency weightings of the major hand–arm substructures [75], which were normalized with respect to the maximum value of the total VPA frequency weighting. They were derived from the substructure VPAs calculated using the model shown in Figure 6a. Because the total VPA is equal to the sum of the distributed VPAs, the sum of the relative VPA weightings is equal to the total VPA weighting. Because the VPA is likely to be correlated with vibration sensation, the features shown in Figure 7b suggest that vibration sensation contours in the low-frequency range (<16 Hz) are primarily due to vibration sensation in the upper arm and shoulder; the dominant sensation location shifts to the forearm, wrist, hand, and fingers sequentially with increases in the vibration frequency. These predictions have been partially confirmed from the findings of an experimental study [99], which investigated the relationship between the substructure VPA and the local vibration perception. Therefore, similar to the total VPA frequency weighting, the standard frequency weighting may approximately be the sum of the relative frequency weightings of the substructure sensations. If the frequency dependencies of the vibration health effects or disorders in each substructure are similar to the frequency dependency of the vibration sensation, it is reasonable to use the standard frequency weighting as a global weighting for the entire hand–arm system for assessing the risk of HTV exposures. This supports the use of the current standard frequency weighting for general control of HTV exposures.

It is, however, not reasonable to use the standard frequency weighting for quantifying finger vibration exposures for assessing the risk of finger disorders or VWF. As shown in Figure 7(b), the trend of the standard weighting is quite different from those of the finger VPA frequency dependency in the low and middle-frequency range. These differences suggest that the standard weighting method may overestimate the prevalence and/or latency of VWF among the workers using low-frequency tools but may underestimate those among workers using tools that generate a large amount of high-frequency vibration components. These predictions are consistent with the findings of several epidemiological studies [100–107]. Unfortunately, the standard frequency weighting was used to quantify the vibration exposure dose for forming the VWF dose–effect relationship adopted in the standard. This mismatch suggests that the adopted dose–effect relationship may not be generally applicable. An important step has been made toward resolving this issue; an alternative finger frequency weighting has been recommended to assess the risk of VWF in an ISO Technical Report [108]. This alternative weighting is very similar to a preliminary finger biodynamic frequency weighting proposed by NIOSH researchers [18]. Further studies are required to test and improve the finger frequency weighting.

#### 4.3.4. Substructure Vibration Power Absorption (VPA) and Average VPA Density

The VPA flowing into the fingers (VPA_Fingers_) can be directly measured or estimated using the real part of the finger mechanical impedance (MIR_Fingers_) and the tool vibration velocity or VPA_Fingers_ = MIR_Fingers_·|A_Tool_/(2π*f*)|^2^ [75]. If it is primarily absorbed by the fingers, the average finger VPA density (AVPAD_Fingers_) can be crudely estimated from AVPAD_Fingers_ = VPA_Fingers_/VOL_Fingers_ = MIR_Fingers_·|A_Tool_./(2π*f*)|^2^/VOL_Fingers_ [109], in which VOL_Fingers_ is the volume of the fingers. The biodynamic frequency weighting of this exposure measure is ${\text{W}}_{\text{Fingers AVPAD}}=\sqrt{{\text{MIR}}_{\text{Fingers}}}/\left(2\pi f\right)$.

The direct estimation method may overestimate the finger power absorption in the low-frequency range because the vibration power can be transmitted from the fingers to the other parts of the hand–arm system in this frequency range. A computer model can be used to separate the VPA absorbed in the fingers from that flowing into the fingers. The model shown in Figure 6a has been used for the estimations of the VPAs distributed in the major substructures of the hand–arm system [75]. The VPA may be assumed to be primarily absorbed in the soft tissue of each substructure. The average VPA density in the soft tissue can be estimated when the volume of the soft tissue in each substructure can be measured or estimated.

#### 4.3.5. Time-Domain Methods

In addition to the root-mean-square (RMS) value conventionally used to quantify the human vibration exposure, the vibration measures in the time domain should also be explored. For example, the RMS value may not be suitable for studying the vibration effects resulting from shocks or impulsive vibrations. This issue can be resolved using a peak counting method widely used in the fatigue analysis of engineering structures to quantify vibration exposure. This requires measuring the vibration in time-history and filtering the data with the desired frequency weighting before counting the peaks. Little research in this aspect has been published [110]. It is unknown which biodynamic response has the best association with specific health effects. Each type of substructure-specific frequency weighting can be used to quantify the exposure and to examine its correlation with various health effects in further studies.

### 4.4. Finite Element Modeling and Applications

**Figure 8.**
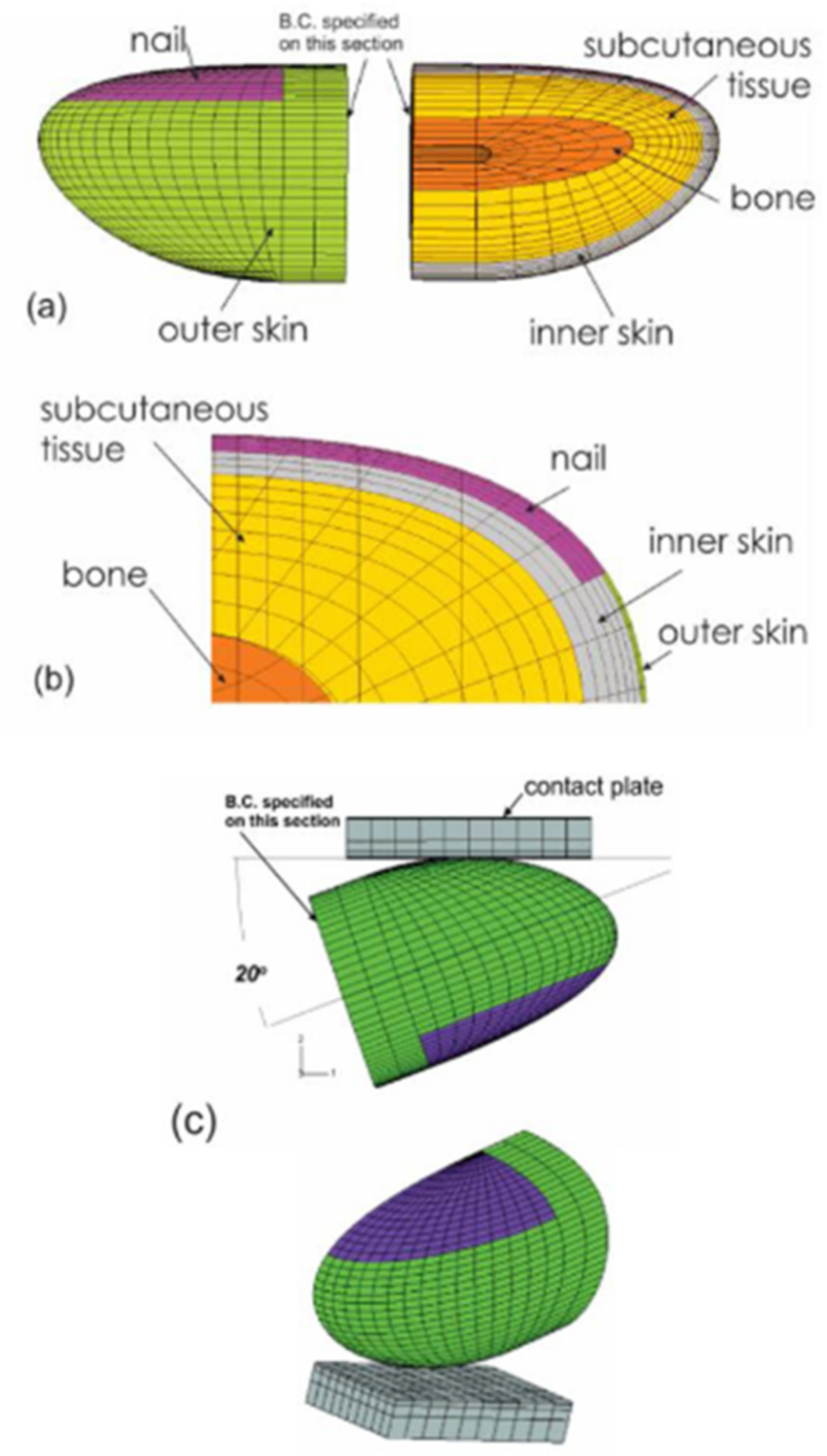
The three-dimensional finite element model of a human fingertip [116,117]. (**a**): External view and longitudinal cross-section. (**b**): Detailed substructures of the model. (**c**): The modelling of the fingertip in contact with a flat surface.

Analysis of the stress and strain of the fingers in response to vibration can help to understand the mechanisms contributing to the development of HAVS. Because the mechanical stimuli on the soft tissues cannot be evaluated experimentally, FE-based biomechanical models of human fingertips were applied to analyze the effects of vibration exposure on the dynamic distributions of stress/strain in the tissues (Figure 8). Macroscopically, a fingertip is composed of skin layers (epidermis and dermis), subcutaneous tissue, bone, and nail. The biomechanical properties of the skin and the subcutaneous tissues influence the transmission of mechanical vibration at different frequencies. Early nonlinear FE models of fingertips were two-dimensional (2D) [111–115], and they have been generalized to three-dimensional (3D) models (Figure 8) [116,117]. The FE models have been applied to several practical problems, such as static and dynamic contact between the fingers and different objects, the biomechanics of the two-point discrimination threshold, and vibration perception threshold tests.

#### 4.4.1. The Time-Dependent Mechanical Response of the Fingertip Subject to Dynamic Loading

Many occupation-related disorders in the hand and fingers are believed to be associated with the local contact pressure between the fingers and the tool handle. The contact interactions between the fingers and handle may also interfere with grasp stability, thereby affecting manipulations of hand-held tools. The time-dependent deformation behaviors of the soft tissue were investigated by imposing different magnitudes of ramp-like loading of the fingertip with different ramping periods and sinusoidal vibrations of the contacting plate at frequencies of 1 Hz and 10 Hz [111]. The models have been used to analyze the time-histories of the tissue displacement at different depths within the fingertip subjected to cyclic loading. Simulations of fingertip/keypad interaction during key tapping have been performed using similar 2D FE models [112–114]. The predicted time-histories of the force responses using the 3D FE model agree well with the corresponding data for the dynamic contact of the fingertip with the flat surface [114]. The time-dependent response indicates that the finger contact stiffness and damping value may change with time during the operation of a vibrating tool, which may affect the vibration transmission and power absorption in the fingers.

#### 4.4.2. Probe/Fingertip Interaction in Vibrotactile Perception Threshold Testing

Vibrotactile perception threshold measurements have been widely used to diagnose the severity of peripheral neuropathy associated with HAVS [118] and sensory loss in stroke [119] and diabetic patients [120]. The vibration perception threshold is believed to be influenced by many factors, especially the finger contact force and vibration frequency [121]. Simulations were performed on the interaction between the fingertips and probe during vibrotactile perception threshold tests using the FE model [112]. The time-dependent deformation profile of the skin surface, strain distributions within soft tissue, and response force of a fingertip were estimated when the fingertip was stimulated using a probe vibrating with a sinusoidal movement. The model predicted the separation between the probe and skin surface during the vibrotactile tests, which is consistent with the experimental data. The simulation results suggest that the fraction of time over which the skin separates from the probe during vibration increases with increasing vibration frequency and amplitude and decreases with increased pre-indentation of the probe. The pre-indentation of the probe has been found to significantly reduce the trend of skin/probe decoupling. The predicted variations of the skin profile as a function of indentation and vibration frequencies compared well with the published experimental data [122].

#### 4.4.3. Simulation of Two-Point Discrimination Threshold Test

**Figure 9.**
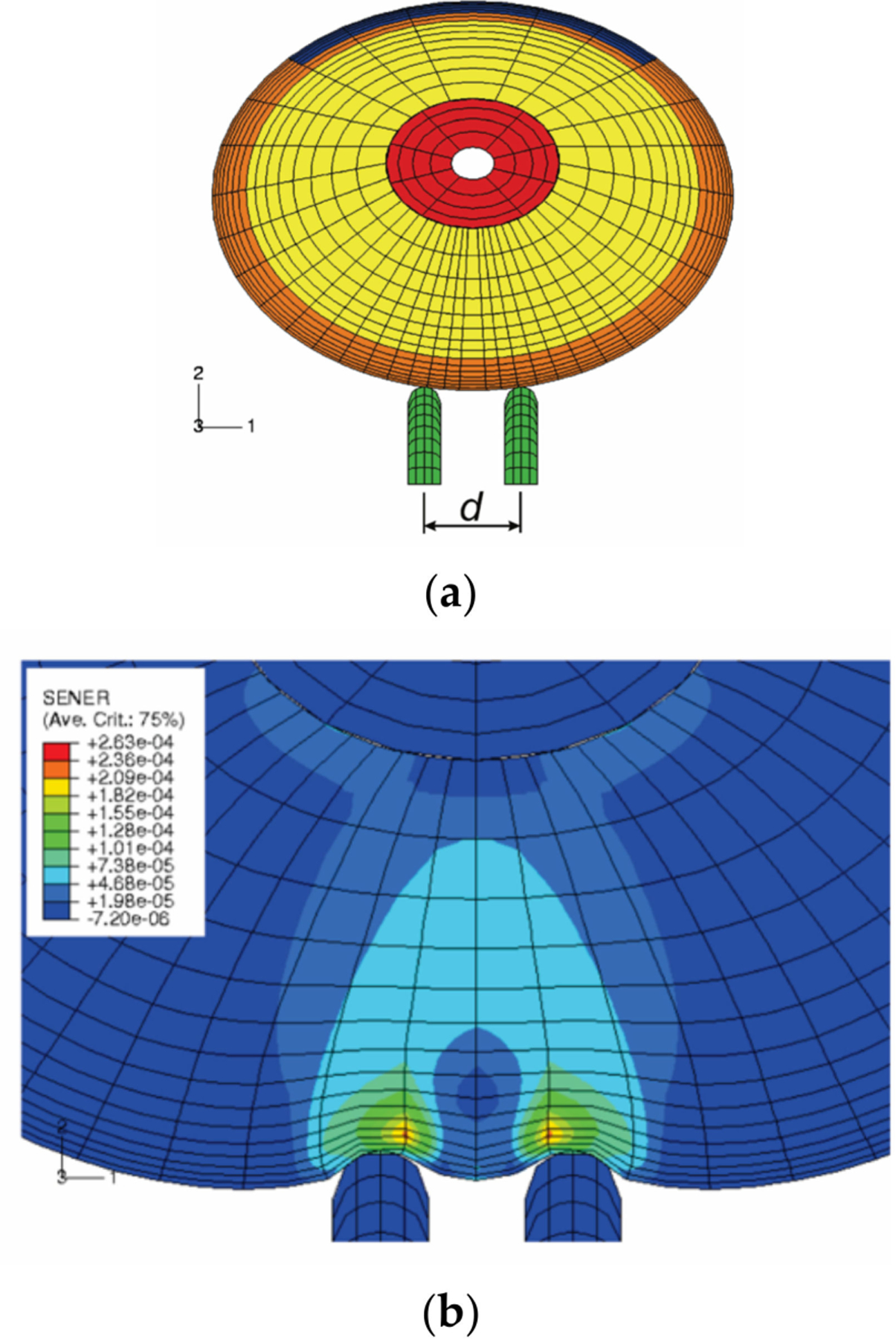
Finite element (FE) simulation of two-point discrimination threshold tests [125]: (**a**) FE model; (**b**) The predicted distributions of the strain energy density (SENER, mg/mm^3^) within the soft tissues of the fingertip indented by the two probe pins.

The tactile sensation of the human fingertips has been widely used for the assessment of health and function in persons who have prolonged exposure to hand-transmitted vibration and carpal tunnel syndrome [121,123,124]. The finger tactile sensitivity depends upon activation of the sensory receptors in the finger skin and transmission of the sensory signal to the central nervous system by sensory nerves. Therefore, tactile spatial resolution in the fingertip is an important factor in the design of vibrotactile arrays. The two-point discrimination distance is used as a measure of tactile spatial resolution. We simulated the biomechanics of tactile sensation using a FE model, as shown in Figure 9) [125]. The mechanoreceptors within the soft tissues were assumed to sense the mechanical stimuli during the tests. The mechanical states (stress/strain) of the tissue at a depth of 0.75 mm from the undeformed skin surface, where the Merkel cell receptors are located, were analyzed. Assuming mechanoreceptors in the dermis sense the stimuli associated with normal strains and strain energy density rather than those associated with shear strain, the theoretical analysis indicated that the threshold of the two-point discrimination test for the fingertip might lie between 2.0 and 3.0 mm, which is consistent with the experimental observations by Perez et al. [126], who reported an average two-point discrimination distance of 2.1 mm during tactile sensation threshold tests of the index finger.

#### 4.4.4. Vibration Modes and Vibration Penetration into the Soft Tissues of a Fingertip

**Figure 10.**
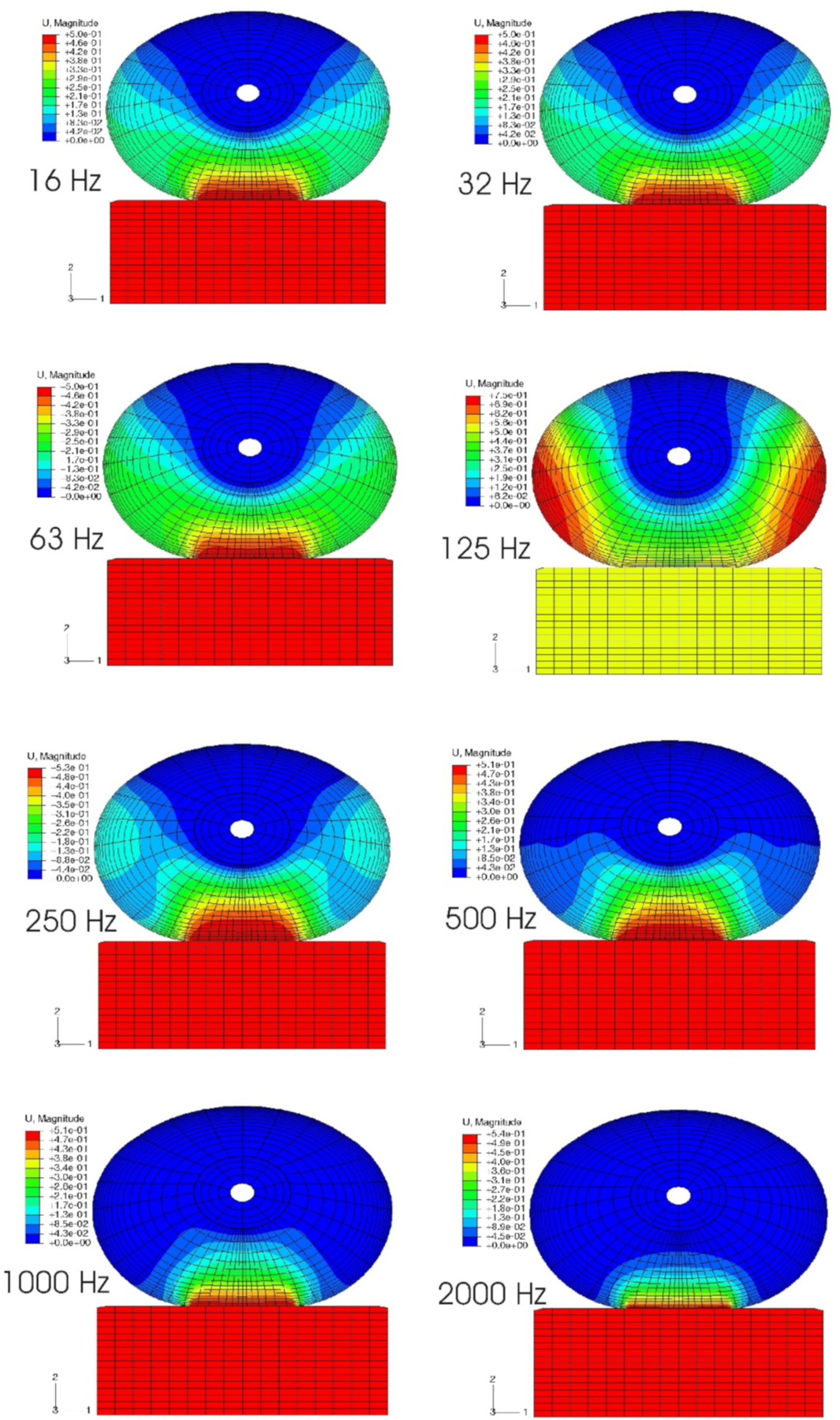
The model predictions of the distributions of vibration magnitude (U, Magnitude, mm) for eight different vibration frequencies (f = 16, 32, 63, 125, 250, 500, 1000, and 2000 Hz) [127]. The fingertip is pre-compressed by 1 mm before being subjected to harmonic vibrations.

The effects of mechanical vibration on the neural and vascular structures in the fingertip are believed to be highly frequency-dependent: low-frequency vibration can transmit from the fingers to the arm and shoulders, while high-frequency vibration will be absorbed in the local soft tissues in the fingers. However, this assertion has never been strictly validated experimentally since the in vivo distributions of the dynamic stress/strain within the fingertip in response to vibration have not been quantified due to technical difficulties. The responses of the fingertip to vibration have been analyzed in the frequency domain using 2D and 3D FE models [115,127,128]. The fingertip was assumed to undergo small harmonic vibrations around the deformed, stressed state, and the perturbed solutions were calculated using the tangential stiffness in the deformed state. Due to the nonlinearly elastic properties of the soft tissue and the geometric nonlinearities, which are accounted for in the static pre-compression process, the tangential stiffness of the soft tissues in the fingertip is location- and pre-deformation dependent. The effect of the pre-compression on the resonant characteristics of the finger has been analyzed using a 3D FE model. The simulation results show that the frequency of the resonant mode associated with the tip tissues depends on the static pre-compression (Figure 10) [127]: the resonant frequency increases from 88 Hz for a pre-compression of 0.5 mm to 125 Hz for a pre-compression of 2.0 mm. Simulation results showed that, at very high frequencies (>1000 Hz), the vibration-induced dynamic strain is primarily concentrated at depths less than 1 mm, and vibration energy dissipates at the skin surface. Although the vibration at very high frequencies may have few acute effects on sensory perception, these mechanical stimuli are well beyond the frequency range of the mechanoreceptors. However, exposure to vibration at very high frequencies may potentially result in structural damage of the local tissues [129,130]. These simulations supported the assertion that low-frequency vibration can transmit from fingers into the body while high-frequency vibration will be absorbed primarily in the local soft tissues near the contact interface.

#### 4.4.5. The Effects of Shear Vibration to Soft Tissues

The vibration strains in any structure include two components: the normal strain as a measure of the deformation in the normal direction and the shear strain as a measure of the deformation in the tangential direction. It is common knowledge that the shear strain is usually more directly associated with the damage of the structure. We analyzed the frequency- and deformation-dependent dynamic strains in the soft tissues in the fingertip that is subjected to vibrations in a direction normal or tangential to the contact surface [131]. Our simulations showed that patterns of the vibration modes and the major resonances for shear vibration are similar to those for normal vibration. Shear vibration induces significant shear strains and negligible normal strains in soft tissue, while normal vibration induces both normal and shear strains in the tissues. The combined normal and shear strain induced by normal vibration may explain why exposure to uniaxial vibration is so damaging to both neural and vascular tissues [132]. Furthermore, the shear strain caused by normal vibration is significant only in the superficial skin layer (<0.3 mm) and negligible deep in the tissue. The shear strains in the superficial layer caused by both normal and shear vibrations have been observed to increase dramatically for vibration frequencies above 250 Hz. Shear stresses may cause significant damage to the skin tissues. However, because the shear strains are concentrated in the superficial skin layer, they may be effectively reduced by using a suitable protective glove.

#### 4.4.6. Response of Mechanoreceptors to Vibratory Stimuli

Exposure to vibration can result in a temporary increase in the vibration perception threshold. The acute effect may result in long-term health effects. Therefore, temporary threshold shifts (TTS) have been used as a laboratory approach to investigate the exposure-response relationship by many investigators [133,134]. We analyzed the frequency-dependent dynamic strains or deformations in the soft tissues surrounding the Meissner’s and Pacinian corpuscles during vibration [127]. The model predictions indicate that vibration exposure in a frequency range from 63 to 250 Hz will induce excessive dynamic strain in the deep zone of the finger tissues, effectively inhibiting the high-frequency mechanoreceptors; while the vibration exposure at low frequency (less than 31.5 Hz) tends to induce excessive dynamic strain in the superficial layer of the tissues, inhibiting the low-frequency mechanoreceptors. The model predictions on the frequency-dependent sensory reduction of the mechanoreceptors following the vibration exposures are consistent with the published experimental observations.

#### 4.4.7. Interaction between Grip Force and Vibration Transmissibility

**Figure 11.**
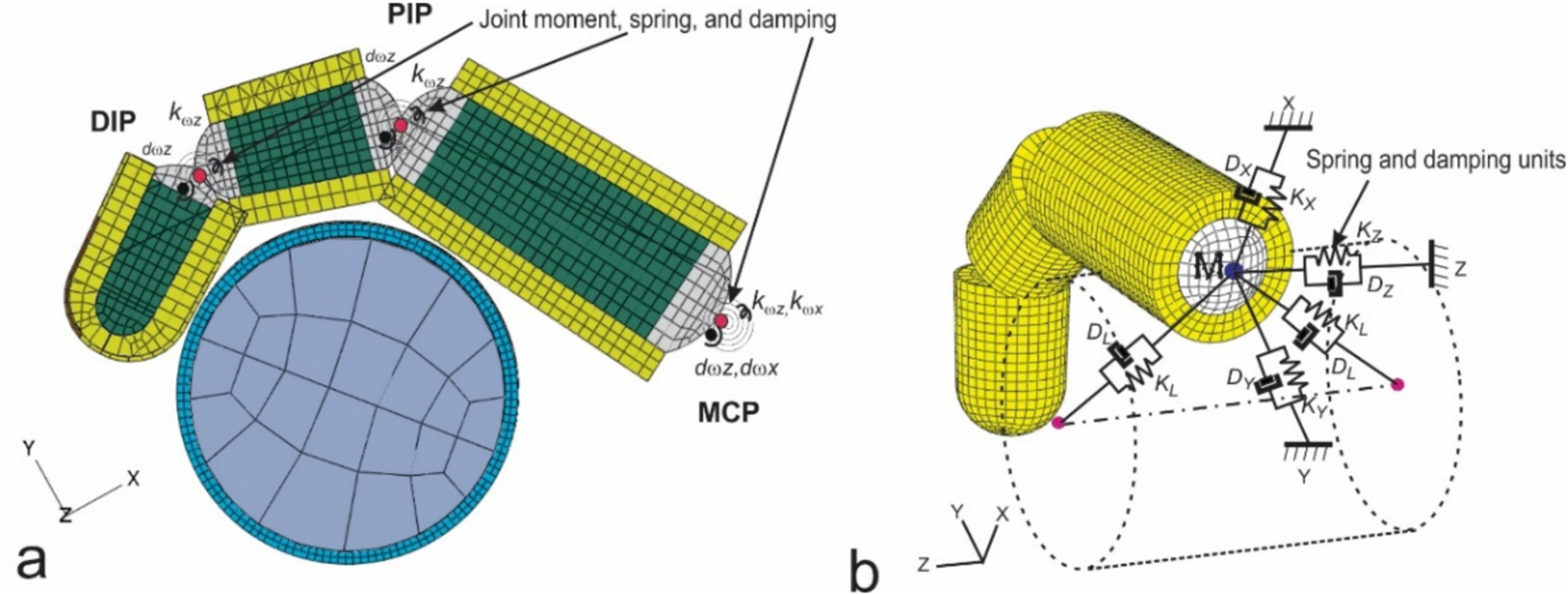
A 3D finite element model of a finger gripping a cylindrical handle [117]: (**a**) The connection of the finger segments by rotational connective elements at the DIP, PIP, and MCP joints; and (**b**) The additional translational, connective elements at the MCP joint point.

It is known that the vibration characteristics of the fingers and hand and the level of grip action interact when operating a power tool. We simulated the vibration of the hand-finger system when gripping a vibrating handle covered with soft materials using a hybrid model (Figure 11) [117]. The hybrid finger model combines the characteristics of conventional finite element (FE) models, multi-body musculoskeletal models, and lumped mass models. This model predicted the local vibration behavior of the finger at each tissue level, while taking into account the effects of the active musculoskeletal force, the effects of the contact conditions on vibration, and the global vibration characteristics. The general trends of the model predictions agree well with the previous experimental measurements in that the resonant frequency increased from proximal to the middle and to the distal finger segments for the same grip force, that the resonant frequency tends to increase with increasing grip force for the same finger segment, especially for the distal segment, and that the magnitude of vibration transmissibility tends to increase with increasing grip force, especially for the proximal segment.

#### 4.4.8. Biodynamic Interaction between the Fingertip and Probe in the Vibrotactile Tests

**Figure 12.**
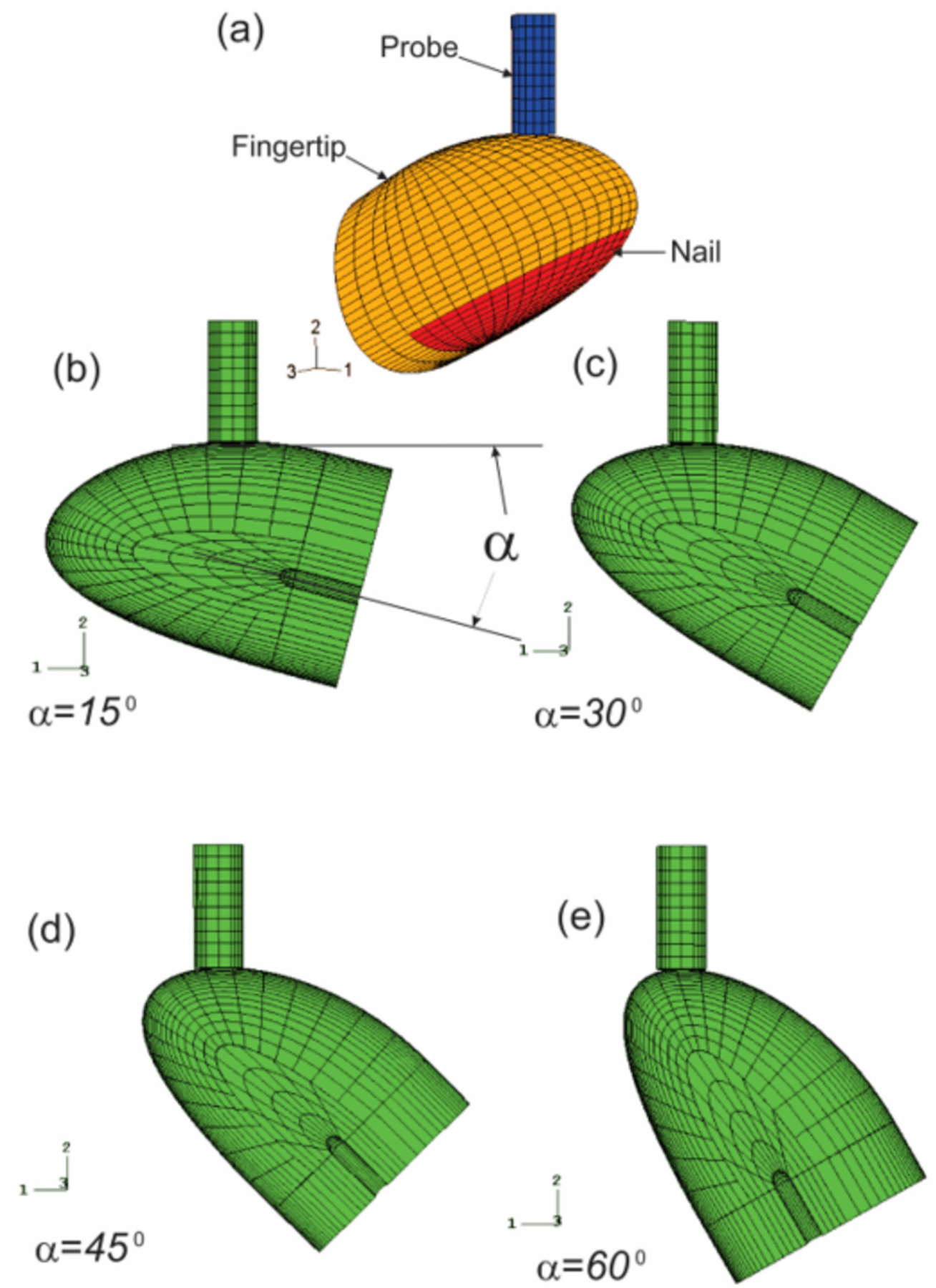
3D FE modeling of the interaction between the probe and fingertip [116]: (**a**) The model; (**b**–**e**) The illustrations of the perspective views of the model with the fingertip is contact with the probe at angles of: (**b**) at 15°; (**c**) at 30°; (**d**) at 45°; and (**e**) at 60°.

Vibrotactile thresholds at the fingertips are affected by several individual, environmental, and testing factors. We analyzed the effects of the contact orientation of the probe on the fingertip and the static pre-indentation on the dynamic deformation of the soft tissues of the fingertip in the vibrotactile tests using a nonlinear finite element model (Figure 12) [116]. The fingertip is contacted by the probe at four different contact locations, which are regulated by contact angles (15°, 30°, 45°, and 60°), and three different pre-indentations (0.5, 1.0, and 1.5 mm).

The model predictions indicated that the average spatial summation of the vibration displacement (SVD) at the fingertip depends on the static pre-indentation and the probe/indenter contact orientation; although the resonance characteristics of the fingertip are not affected by either the pre-indentation or the contact location. The location dependence of the vibration exposure factors at the fingertip was found to increase with increasing static pre-indentation. At a static indentation of 1.5 mm with the test conditions specified in ISO 13091–1 [121], the values of the SVDs determined at different probe/fingertip contact orientations differ as much as 125%. Since the dynamic displacements of the soft tissues are believed to affect the vibrotactile threshold, the simulation results suggest that the contact orientation of the probe on the fingertip should be strictly defined and restricted to obtain reliable results in the vibrotactile perception threshold tests.

#### 4.4.9. Further FE Model Development

No finite element model of the entire hand–arm system has been reported. In future studies, there is a need to develop such a complex model and apply it to confirm and improve the identified biodynamic frequency weightings for the major substructures of the hand–arm system. The complex simulations may be used as a basis to develop study designs for the substructure-specific methods for quantifying the vibration exposure and examining the related health effects. To make the FE modeling more efficient but less expensive and less technically demanding, some influencing factors such as the time-dependence and non-linear features of the tissue biodynamic properties may be ignored or considered as a random factor in the analyses, at least in the initial version of the FE model. The coupled handle–hand–arm system under certain hand forces and postures can be directly considered in the model development and simulations. While it is difficult to obtain accurate biodynamic properties of the hand–arm system, the driving point biodynamic response functions and the vibration transmissibility spectra measured at many points on the hand–arm system could be used to calibrate the FE model, similar to those used in the calibration of the lumped-parameter models.

## 5. Influencing Biomechanical Factors and Effect Assessments

Hand forces and hand–arm postures are likely to affect the development of vibration-induced health effects through the following three mechanisms: (i) Increasing the hand forces and changing the hand–arm postures from their neutral positions increase the quasi-static stresses and strains of the tissues in the hand–arm system; the increased stresses and strains are likely to increase injury potential, similar to the effect of the quasi-static stresses on the engineering material fatigue [17]; (ii) the hand forces and hand–arm postures may affect the biodynamic responses of the system [54,68,75,135,136], or the vibration stresses and strains that are super-imposed on the quasi-static components; and (iii) the hand forces and awkward hand–arm postures may also cause adverse physiological responses or effects; for example, an overhead operation may substantially reduce the blood circulation in the hand–arm system; a large hand contact pressure may reduce the blood circulation in the local contact tissues; awkward hand–arm postures may also make the joints and connecting tissues vulnerable. These combined effects may increase the injury potential of the hand–arm system. Major remaining research should examine how to effectively quantify these factors at workplaces and include them in risk assessment.

Because it is difficult to instrument each tool handle to measure the hand forces during the tool operations, a practical method for force measurement is to apply the hand forces perceived in a tool operation on a separate nearby instrumented handle, which is termed as the force matching method. We examined this method [56,57]; the results suggest that vibration exposure is likely to reduce the matching accuracy, but it could provide a reasonable approximation of the forces applied in the operations of some vibrating tools.

Another approach for measuring the hand forces is to use an instrumented glove equipped with contact pressure sensors [43]. To apply this approach, the relationships among hand forces were examined and the characteristics of the contact pressure distributions were identified [137,138]. We proposed a novel theory for characterizing the grip force [139]: the grip force can be approximately simulated using an elliptical function. This theory demonstrates that the measurement of grip force is orientation-specific; the maximum difference among the measurements at different orientations can be up to 40% on a 40 mm handle [139]. To avoid this issue, it is better to quantify the grip force by measuring the total contact force on a handle. This led to the invention of a novel dynamometer for reliably measuring the total grip force and characterizing the grip force distributions around the handle [140]. This novel grip dynamometer has been used to evaluate the effects of handle size and gloves on grip strength [141,142]. These studies have also helped the development and improvement of the standard on hand force measurements [43].

We also investigated the effects of hand forces on biodynamic responses [55,75]. These studies resulted in the following conclusions: (i) increasing the hand forces generally increase the biodynamic responses; (ii) the hand force effect on vibration transmissibility of the hand–arm system is location- and frequency-specific; (iii) at each location for a given vibration frequency, the hand force effect becomes nonsignificant when the hand force is beyond a certain value; and (iv) increasing the push force may reduce the finger vibration response in the low and middle-frequency range. Such knowledge may be used to help determine how the hand forces can be considered in risk assessments of HTV exposures.

The reported studies have clearly demonstrated that the biodynamic responses of the fingers are primarily affected by the force applied by the fingers [66]. Furthermore, the effect of the fingers-applied force on the finger biodynamic responses is different from the effect of the hand palm-applied forces on the biodynamic responses in the palm–wrist–arm substructures. For example, increasing the push force may reduce the finger responses in the low and middle-frequency ranges but it generally increases the responses in the palm–wrist–arm substructures [66]. Hence, it is not appropriate to use the hand coupling force (grip + push) as a weighting factor for assessing finger disorders or VWF, similar to the mismatch between the hand frequency weighting and VWF. It may be more appropriate to use the grip force as a basis to determine the force weighting factor for assessing finger disorders and to use the coupling force as the basis to determine the force weighting factor to assess the risk of HTV exposures for the remaining hand–arm system substructures. Determination of the specific force weighting factors remains an issue for further studies. The force weighting factors may be determined based on the information in the following four aspects: (i) the effects of the grip force or hand coupling force on vibration injuries that may be identified from experimental studies using an animal model; (ii) the effects of the force on the biodynamic frequency weightings for different substructures; (iii) the effects of the force on the location-specific vibration psychophysical effects; and (iv) the effects of the force on the biological factors or properties.

## 6. Intervention Methods and Technologies for Controlling Hand-Transmitted Exposure

One of the major approaches for controlling vibration exposure is to minimize the vibrations on tool handles or handheld workpieces without reducing the efficiency of the work. This requires optimizing the designs of tools and vibration-reducing (VR) devices. Because the behaviors of a vibrating tool or VR device are usually affected by the biodynamic properties of the hand–arm system, another major aim of biodynamic studies is to provide reliable biodynamic information of the system to help analyze and design tools and VR devices. For this reason, an international standard has been established to recommend representative biodynamic data and models of the hand–arm system [41]. We substantially improved this standard by updating its mechanical impedance data and biodynamic models [49,76].

Besides the modeling studies, we also conducted many experimental studies for testing and evaluating powered hand tools [143–150], which have been used not only to develop or improve the tool test standards but also to help select tools. NIOSH researchers have developed a dataset that includes vibration emissions of many hand-held powered tools (https://www.cdc.gov/niosh/topics/noise/noise_levels.html (accessed on 9 June 2021). They have also helped develop a standard for helping select the tools [151,152].

**Figure 13.**
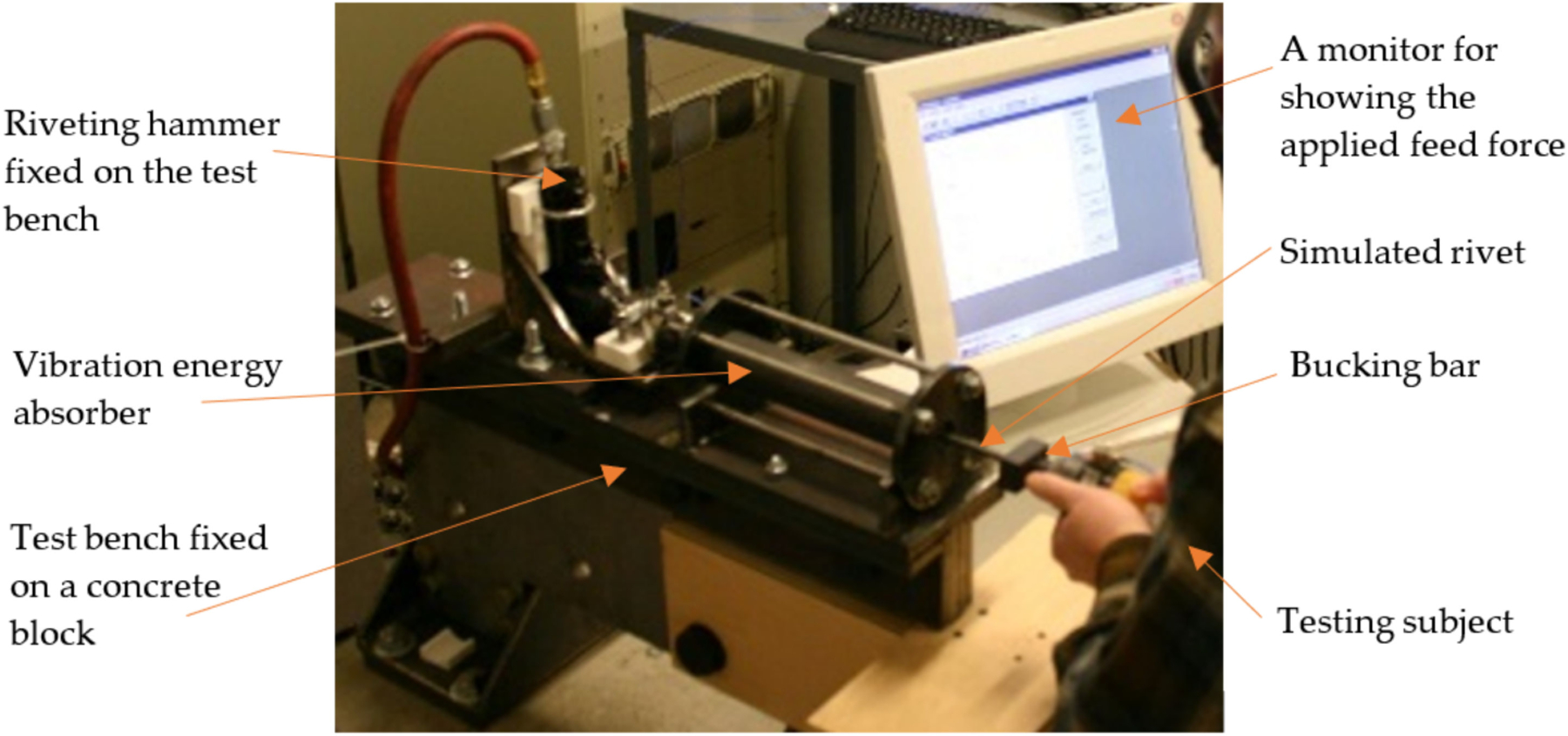
A proposed bucking bar test rig [150], which is composed of the following components: (i) A remote-controlled pneumatic riveting hammer programmed to deliver consistent vibration stimuli; (ii) An energy absorber for dampening the vibration input to the simulated rivet; (iii) A simulated rivet; (iv) A force plate for measuring the ground reaction force (feed force); and (v) A computer monitor for displaying the applied feed force as a strip chart allowing the bucking bar operator to maintain the target force within the specified range. The tested bucking bar is pressed against the simulated rivet by a test subject. Tri-axial acceleration data are simultaneously collected at the riveting hammer, the bucking bar, and at the right wrist of the bucking bar operator.

The sheet metal riveting process requires not only a riveting hammer but also a bucking bar to apply the necessary opposing force. Bucking bar operators can be exposed to repeated high shocks during riveting operations. As a result, the prevalence of VWF among bucking bar operators may be several times higher than that of the riveting hammer operators [106,153,154]. It is very important to control the bucking bar vibration. Because bucking bars are not powered hand tools, no standard test method has been established. To help evaluate the effectiveness of vibration-reduced bucking bars and related VR devices, a testing method has been proposed [150]; its test rig is shown in Figure 13.

Vibration-reducing gloves are the most convenient VR devices. Their effectiveness has been systematically examined by measuring and modeling the glove vibration transmissibility using both to-the-hand and on-the-hand methods. The related studies include the following aspects: (i) Improving the methods and techniques for measuring the glove vibration transmissibility at the palm of the hand [52,155–158], which increased the accuracy and reliability of the testing results and contributed to a major revision of the VR glove test standard [42]; (ii) Developing a novel method for conveniently and reliably measuring the glove vibration transmissibility at the fingers [35], which may be included in the standard VR glove test in its future revision; (iii) Enhancing the understanding of the glove VR mechanisms and influencing factors through examining the correlation between the glove vibration transmissibility and the mechanical impedance of the hand–arm system [159–161], and developing computer models of the tool–glove–hand–arm system [53,77,162]; (iv) Measuring the glove transmissibility and investigating their influencing factors [44,54,62,163]; (v) Evaluating and applying a transfer function method to estimate tool-specific performance of the gloves [32,62,161,164,165]. The major conclusions made from these VR glove studies are as follows: (i) VR gloves may result in significant adverse effects such as increased hand fatigue and reduced finger dexterity because the gloves can increase the hand grip effort on a tool handle [141]; (ii) The available VR gloves do not usually reduce vibration transmitted to the hands at frequencies below 25 Hz; hence, it is better to use ordinary work gloves when operating low frequency tools such as rammers, tampers, and vibrating forks [164]; (iii) VR gloves can effectively reduce high frequency vibration components and sharp peaks [35,69,164,166]; (iv) Increasing the thickness of the glove cushioning materials and/or the suspended glove mass can increase the cushioning effectiveness of the glove but these changes can also increase the adverse effects of the glove [53,141]; hence, the current criteria for a certified anti-vibration glove require a limited thickness of the gloves; for these reasons, it may be difficult to improve the effectiveness of VR gloves from their current level by increasing their cushioning function; (v) Besides the cushioning function, a VR glove may also affect the finger or hand vibration through the other functions or factors of the glove [77,167]; for example, wearing a tight glove may increase the finger soft tissue stiffness due to the constraint of the glove material around each finger; the increased finger stiffness must affect the finger vibration response or the vibration power absorption distribution in the hand–arm system [77]; the glove may also change the hand grip dimension, finger positions on a handle, and hand contact pattern, which may affect the detailed vibration distribution input to the hand; these factors should also be considered in the optimization design of VR gloves; the combined effect of the cushioning function and other factors can be evaluated using on-the-hand methods [69,77,167,168]; and (vi) Because ordinary work gloves may exhibit some of these additional functions, we hypothesize these gloves may also provide some protection of the fingers and hand during vibration exposure, despite exhibiting little cushioning function. Further studies are required to test this hypothesis.

We, together with our collaborators, have also conducted a series of investigations on handheld workpiece vibrations. First, we measured and characterized the vibration exposure of workers grinding typical handheld workpieces (golf club heads) at a workplace and preliminary strategies/methods for controlling the grinding vibration exposure were proposed [169]. Second, we measured vibration responses of the workpiece–hand–arm system in laboratory experiments [170], and developed a model of the system based on the measured response functions [78], as illustrated in Figure 6c. Third, we evaluated proposed engineering strategies or methods using the developed model [79]. Based on the findings of the studies, several effective engineering intervention methods for controlling handheld workpiece vibration have been proposed. These interventions depend on the specific conditions and requirements at workplaces. Further studies are required to implement the identified methods through developing specific technologies. Bucking bars can be considered a special type of handheld workpiece. The identified intervention methods may also be applicable to help develop more effective anti-vibration bucking bars.

Mechanical arms and exoskeletons have been developed and used to help reduce the burden of the hand–arm system. Our studies found that mechanical arms can marginally reduce the vibration magnitude (about 10%) [149]. Their further developments may help reduce more vibration. However, the use of these devices may increase the exposure time, especially during overhead operations. As a result, the daily vibration exposure dose may be increased, which should be controlled in the implementation of the new technology. An obvious solution is to select low vibration tools for the operation, although the efficiency of the task should be maintained. Another potential engineering solution is to incorporate a vibration-reducing device on the mechanical arm system. The use of a mechanical arm or exoskeleton may allow the use of heavier tools. This may also make it possible to design a heavier tool with lower vibration emissions. Further studies are required to optimize their combined solutions. Further studies should also evaluate the following potential adverse effects of exoskeletons: (1) Any exoskeleton has a certain mass, which may increase not only the static load but also the dynamic load on the body of a worker, especially when exposed to whole-body vibration; and (2) the use of an exoskeleton may increase the vibration transmission.

## 7. Biological Effects of Hand-Transmitted Vibration Exposure

Exposure to HTV via the use of power- or pneumatic-hand tools results in an increased risk of developing cold-induced finger blanching (i.e., vibration-induced white finger) and deficits in neurosensory perception including reductions in vibrotactile sensitivity and touch perception, and alterations in the sensitivity to both cold and warm stimuli by the fingers [1,171–178]. Workers with these symptoms, or HAVS, may also experience reductions in grip strength and manual dexterity, reduced muscle strength in their forearms, and tendonitis in the wrist, elbows or shoulders [179–194]. Although there has been a great deal of research done on workers in various occupations to characterize the effects of working with vibrating hand tools on the risk of developing, and prevalence of HAVS [30,195–198], there are still many questions regarding the etiology of these disorders, and how the various components of vibration (e.g., frequency, amplitude, duration of the exposure) along with other work-related factors (e.g., awkward posture, ambient temperature, and personal health factors) contribute to the risk of developing HAVS [91,199,200]. This section of the review describes what is known about the association between the various exposure factors and the risk of developing symptoms of HAVS along with the description of various models that have been used to characterize exposure-response relationships, potential biomarkers that have been identified for the early detection and/or diagnosis of the syndrome, the effectiveness of anti-vibration materials in reducing or eliminating the effects of vibration exposure, and individual factors that may contribute to the development of the disorder.

### 7.1. Tail Model and Sinusoidal Vibration

#### 7.1.1. Biodynamic Response in an Animal Model of Vibration-Induced Injury

**Figure 14.**
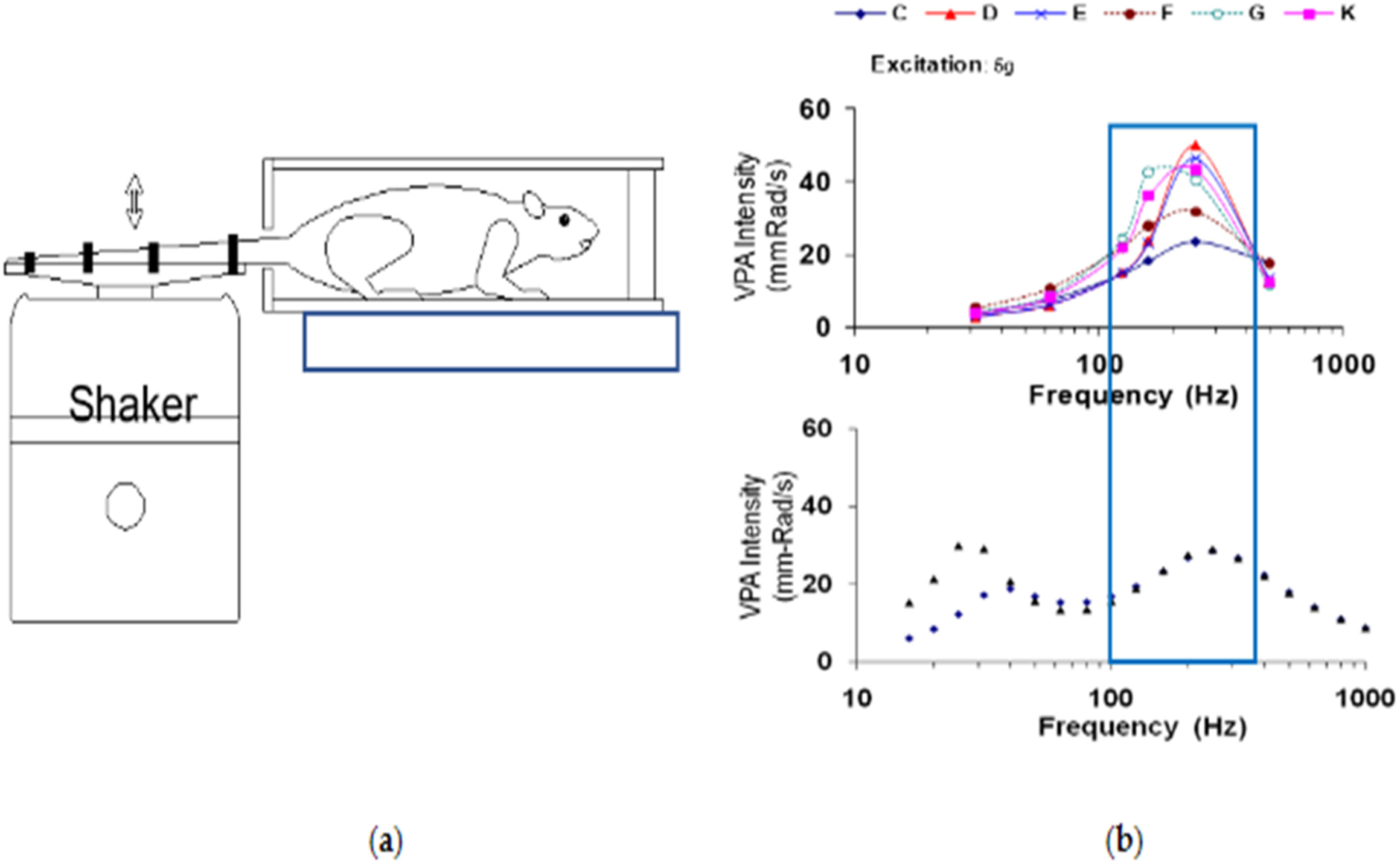
The rat-tail model and the vibration response characteristics of the rat tail on the vibration platform: (**a**) The sketch of the rat-tail vibration exposure system; (**b**) the comparison of the vibration power absorption (VPA) intensity of the rat tail at 6 different points along the length of the tail (points C-K anterior to posterior) for four animals (top figure [205]) with those of the human fingers (bottom figure [75]); they had similar resonant characteristics between 100 Hz and 300 Hz.

To begin to address some of the questions regarding the exposure–response relationship between vibration and injury or dysfunction, we developed and characterized an animal model of vibration-induced injury. This model was designed to determine how various vibration-associated characteristics (frequency, amplitude, and duration of exposures) affect the transmission of the vibration signal to various tissues, and how these factors are associated with the development of soft tissue injury. Because of similarities in size and anatomical structure, a rat-tail model of vibration-induced injury was previously developed to determine the mechanisms that might underlie vibration-induced injury to the fingers [201–204]. This model was refined and characterized by the NIOSH researchers [67,166,205] and has been used to determine how vibration frequency affects the risk of developing certain symptoms of HAVS and to provide information regarding the etiology of the disorder. This model involves restraining male rats in Broome-style restrainers and securing the tail to a platform attached to a shaker, with four 1 cm wide elastic straps that are placed along the length of the tail, as illustrated in Figure 14a. To characterize the biodynamic response of the tail to vibration, a laser vibrometer was used to measure the amplitude of the tail in response to vibration at 8 different locations, 6 different sinusoidal frequencies (32 Hz, 63 Hz, 125 Hz, 160 Hz, 250 Hz, and 500 Hz) and 3 different accelerations (9.8, 49 and 98 m/s ^2^ root mean squared) [205]. The amplitude of the tail under various exposure conditions was divided by the amplitude of the platform to calculate transmissibility (i.e., the biodynamic response). This study demonstrated that physical responses of the rat tail were similar to those of the human finger [45,54,64]; Although overall transmissibility was higher in the rat tail, the resonant frequency range of the rat tail was in the same range as that of the human fingers, as shown in Figure 14b, i.e., 100–300 Hz, depending on the location of the measurement. The changes in acceleration resulted in minor shifts of the resonant frequency of both the fingers and tail at frequencies greater than 60 Hz [205].

#### 7.1.2. Frequency-Dependence of Vibration-Induced Vascular Dysfunction

**Figure 15.**
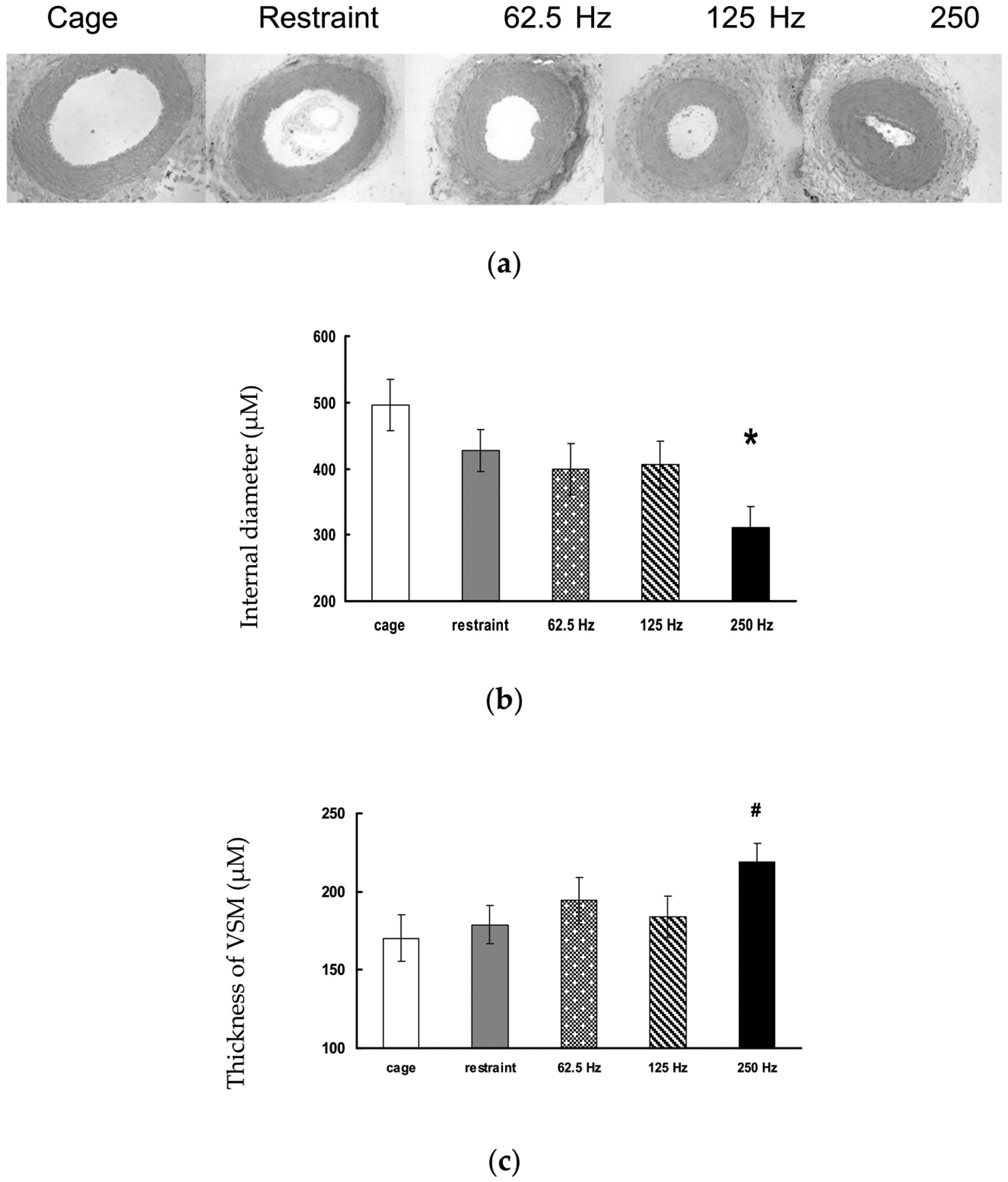
(**a**) The photomicrographs show hematoxylin and eosin staining in the ventral tail arteryafter exposure to vibration at various frequencies; (**b**) Exposuer at 250 Hz resulted in a significant reduction in the internal diameter of the ventral tail artery (* different than all other conditions, *p* < 0.05); and (**c**) An increase in the smooth muscle thickness in the ventral tail artery (# different than cage control, *p* < 0.05).

Based on the findings of the biodynamic study, the rat tail model was used to examine the frequency-dependent effects of vibration exposure on vascular and sensorineural function. Initial studies examined the effects of exposure to vibration at 62.5, 125 and 250 Hz (49 m/s^2^ rms). These frequencies were examined because transmissibility (or the biodynamic response) of the mid-portion of the tail was at unity at 62.5 Hz (1:1 with the platform), but displayed increasing resonance at 125 and 250 Hz [205]. This allowed us to test the hypothesis that injury or dysfunction would be increased with exposure to vibration frequencies that induced a greater biodynamic response (or resonance). Vascular responses to vibration were frequency-dependent; although exposures at all frequencies resulted in an increase in oxidative stress, anti-oxidant enzyme concentrations, inflammation and cellular factors that play a role in vascular remodeling, morphological measures of the ventral tail artery demonstrated that vibration exposure at frequencies that generated greater resonance of the tissue (62.5 < 125 < 250 Hz) resulted in a reduction in the internal diameter of the artery, a thickening of the vascular smooth muscle of the artery (Figure 15), and an increased expression of a marker indicative of oxidative-induced tissue damage, nitrotyrosine [206]. This increased and maintained vasoconstriction is similar to that seen in biopsy samples collected from the fingers of workers with HAVS [207]. Studies examining the effects of vibration on factors mediating vasoconstriction and dilation have demonstrated that exposure to a single bout of vibration at 125 Hz results in an increased sensitivity of the tail artery to the α2c-adrenoreceptor agonist, UK14304, and reduced sensitivity of the tail artery to the vasodilating effects of acetylcholine [ACh [208]].

Additional studies have demonstrated that the reduced sensitivity of the artery to ACh after exposure to a single bout of vibration persists for at least 8 days following the exposure [209]. ACh stimulates the release of nitric oxide from endothelial cells, and the vibration-induced reduction in the sensitivity of arteries to changes in ACh-induced vasodilation appears to be due to a decrease in nitric oxide concentrations in these arteries [209]. This suggests that vibration exposure at or near the resonant frequency of the tissue may result in prolonged vasoconstriction due to changes in responsiveness to endogenous vasoconstricting and vasodilating factors. These findings are also consistent with the results of other experimental and epidemiological studies suggesting that the current frequency weighting in the ISO-5349 [4] may need to be revised to take into account the biodynamic responses and biological effects of exposure to vibration frequencies greater than 60 Hz [54,75,210].

The effects of coupling between the vibrating source and the tail were also examined. In the initial studies, four straps were used to secure the tail. However, the significant increase in the amplitude of the tail response (as compared to the response in human fingers [54,75]) in the resonant frequency range suggested that there was a reduction in coupling at the resonant frequency [205]. To determine if the peripheral vascular and sensorineural effects were still amplified at the resonant frequency, coupling between the tail and vibrating platform was measured using 4 straps as in the previous study, or 7 straps to restrain the tail and increase coupling. To reduce the number of animals used in the study, vibration at 62.5 Hz (a frequency that does not induce resonance) was compared to 250 Hz (a frequency that induces resonance). The results of these studies demonstrated that exposure to 250 Hz (49 m/s^2^ rms) resulted in a significant reduction in the internal diameter of the ventral tail artery and an increase in the thickness of the vascular smooth muscle after 10 consecutive days of exposure with 7 straps, as compared to arteries from animals whose tails were restrained with 4 straps (there were similar morphological changes with 4 straps, but the variability was greater). Exposure to vibration at 250 Hz also resulted in an increase in gene transcription of the antioxidant, metallothionein-1a, the extracellular matrix protein intracellular adhesion molecule-1, and the immediate early gene, myeloid leukemia-1 protein when both 4 and 7 straps were used for restraint. However, the increased expression of these transcripts was significantly greater when 7 straps were used for restraint instead of 4. The increased expression of these genes may initially be involved in the growth of new arterioles and capillaries to increase perfusion of the surrounding tissue when the ventral tail artery is constricted for a period of time [211–213]. However, with years of exposure to HTV, the generation of new arterioles may become pathological and result in the development of tortuous blood vessels often found in the fingertips of workers with VWF disease [207,213,214]. Vibration at 62.5 Hz resulted in a reduction in cyclo-oxygenase_2_ (*cox*_*2*_*)* gene expression when both 4 and 7 straps were used for restraint of the tail, and a reduction in *cox*_*2*_ restraint control animals when 7 straps were used. *Cox*_*2*_ is involved in mediating vasodilation and enhances inflammation through the prostaglandin pathway. Exposure to vibration at 250 Hz resulted in an increase in the transcription of this gene, but it was not associated with vasodilation. Instead, it may have acted as a signaling factor and stimulated an increase in prostaglandin synthesis [215]. Taken together, the results of this study suggest that exposure frequency and coupling between the appendage and the vibrating sources affect the biological responses. These findings are also consistent with those of the experiment using only 4 straps for restraint, which shows that although there are changes in markers of vascular dysfunction at all vibration frequencies, the changes in these markers are more dramatic and occur more quickly in response to vibration exposure at or near the resonant frequency.

#### 7.1.3. Frequency-Dependence of Vibration-Induced Sensorineural Dysfunction

Sensorineural effects of vibration are usually seen before vascular effects, and studies performed using the animal model described above demonstrated that the changes in sensorineural function are frequency dependent. Exposure to vibration for 10 days at 62.5, 125 and 250 Hz resulted in changes in sensorineural function. Sensitivity to transcutaneous electrical stimulation was reduced (i.e., animals were more sensitive) with exposure to all 3 vibration frequencies, but the magnitude of the change in the response was greater with exposure to vibration at 250 Hz. In addition, vibration exposure at or near the resonant frequency (125 and 250 Hz) resulted in a greater reduction in the sensitivity to touch or applied pressure [216]. These changes were associated with an increase in the expression of pro-inflammatory cytokines and factors that mediate oxidative stress, which in turn may have resulted in a reduction in the myelination of nerves and transmission of nerve impulses from peripheral nerves to the central nervous system. Concentrations of specific cytokines were also measured in the circulation; after 10 days of exposure to vibration at 250 Hz, circulating interleukin (IL)-1 concentrations were significantly increased, suggesting that changes in this cytokine may be a marker of vibration-associated injury [216].

The effects of increased coupling on sensorineural function were also assessed by comparing the effects of tail restraint with 4 vs. 7 straps as described above [217]. The effects of vibration on sensory physiology were not measured in this study. However, the effects on gene transcription for factors involved in myelin production, cell signaling through ion channels, and factors that have been associated with the development of chronic pain were measured in nerves and the dorsal root ganglia along with concentrations of the anti-oxidant enzymes, glutathione- (GSH), superoxide dismutase-1 and −2 (SOD1–2) in peripheral nerves. Exposure to vibration at 250 Hz resulted in a significant increase in transcript levels for GTP-cyclohydrolase-1, a gene that has been associated with an increased risk for developing chronic pain, and hypoxia-induced factor-1a, regardless of the number of straps used or restraint. However, the increase in the transcription of these genes was greater when the tail was restrained with 7 than with 4 straps [217]. Myelin-associated glycoprotein (*mag)*, a marker of myelin regeneration, was only increased in tail nerves with exposure to vibration at 62.5 Hz and only when 4 straps were used for restraint. The use of 7 straps for restraint resulted in a reduction in *mag* with exposure to vibration at 250 Hz, but this decrease was not statistically significant. In the dorsal root ganglia (DRG), exposure to vibration at 62.5 Hz resulted in an increase in cyclooxygenase (*cox)*_2_ which may have resulted in an increase in blood flow and inflammation in the DRG, and reductions in the n-tyrosine kinase (*ntrk*) receptor, which mediates the effects of nerve growth factors [218]. These changes occurred when both 4 and 7 straps were used for restraint. Exposure to vibration at 250 Hz resulted in an increased expression of the neurotrophic tyrosine kinase receptor and a reduction in the transient-vanilloid receptor protein-1 (*trpv-1*), an ion channel that is located in nervous system cells that transmit information about peripheral and visceral pain, itch and temperature to the nervous system [219,220]. Restraining the tail with 7 straps significantly increased the expression of these genes in the DRG as compared to restraint with 4 straps in animals exposed to vibration at 250 Hz [217]. Exposure to vibration also resulted in a significant increase in concentrations of the anti-oxidant enzyme, SOD2 in the DRG, but only with exposure to vibration at 250 Hz and using 7 straps for restraint. These findings are consistent with the hypothesis that increasing the coupling between the tissue and the vibrating source will exacerbate the effects of the vibration exposure, and that in most cases, the effects are more prominent when the exposure is at the resonant frequency [217].

#### 7.1.4. Potential Biomarkers of Vibration-Induced Injury

The 10-day exposures demonstrated that there are frequency-dependent effects of vibration on the peripheral vascular and sensorineural systems. Based on the results of these studies, additional studies were designed to clarify which measures could potentially serve as physiological or biological markers of vibration-induced injury. These studies focused on the sensorineural system because changes in peripheral sensory function usually occur prior to vascular effects, and workers are less likely to regain normal sensorineural function, even after they stop using vibrating hand tools [221–224]. Based on the findings of the 10-day study, animals were exposed to 3d of vibration at 62.5 or 250 Hz to further define physiological or biological markers that might be used for early detection of peripheral nerve injury or sensory dysfunction [201]. After 2d of exposure to vibration at 250 Hz, all nerve fibers (unmyelinated C-fibers, myelinate Aδ fibers, and myelinated Aβ fibers) displayed an increased sensitivity to electrical stimulation, indicating that these stimuli were perceived as noxious or uncomfortable. Animals exposed to vibration at 250 Hz also displayed an increased sensitivity to warmth. However, there were no vibration-induced changes in sensitivity to touch or applied pressure with this shorter exposure. Exposure to vibration at both 62.5 and 250 Hz resulted in increased transcript expression of *il*-1β and tumor necrosis factor (*tnf*)-α, and a reduction in lipid peroxidation in the ventral tail nerve. Because the nerves are small, there was not enough tissue to measure both lipid peroxidation and reactive oxygen species concentrations. However, based on the data that were collected, vibration at both frequencies resulted in an increase in the expression of inflammatory factors. This increase in inflammation may have stimulated anti-oxidant activity which in turn reduced lipid peroxidation. In the DRG, nitrotyrosine and glutathione were increased with exposure to vibration at 62.5 Hz, indicating that exposure to vibration at this frequency resulted in an increase in oxidative stress. Vibration-induced changes in the lumbar spinal cord were also measured; exposure to vibration at 250 Hz resulted in a reduction in myelin basic protein, and PSD95, a marker of synapses. Exposure to vibration at both 62.5 and 250 Hz resulted in an increase in *tnf-α* in the spinal cord [201]. These findings suggest that exposure to vibration at the resonant frequency resulted in a reduction in myelin production and synapse number or function in the spinal cord. These changes may also have reduced the transmission of the vibrating signal from the periphery to the central nervous system. Exposure to vibration at 250 Hz also resulted in an increase in circulating IL-1β concentrations. Because vibration at 250 Hz increased concentrations of this peptide with both a 3 and 10-day exposure, it may serve as a marker of vibration-induced injury.

To determine how a longer-term exposure affected sensorineural function, a 28d exposure to tail vibration at 125 Hz (49 m/s^2^) was performed. Physiological measures were collected prior to exposure on days 1, 7, 14, 21 and 25 of the study [225]. As in previous studies, exposure to vibration within the resonant frequency range initially resulted in reduced sensitivity of large myelinated Aβ-fibers to transcutaneous electrical stimulation. However, after 24d of vibration exposure, there was a reduced sensitivity of Aβ fibers to transcutaneous electrical stimulation. These findings are consistent with data collected from workers diagnosed with HAVS [226]. Quantification of myelin staining using the histological stain, toluidine blue, showed that both restraint and exposure to vibration for 28 d resulted in an increase in myelin disruption. When immunohistochemistry was performed on peripheral nerves, there was a vibration-induced reduction in 3′4′-cyclic-nucleotide phosphatase staining (a marker of myelin–nerve fiber interactions) and myelin basic protein staining as compared to unexposed and restraint-exposed animals [225]. Because peripheral nerves mediating pain and peripheral vascular function can be identified using immunostaining for calcitonin-gene-related peptide (CGRP), levels of immunostaining for this peptide were assessed in the DRG. Twenty-eight days of vibration exposure resulted in a significant reduction in CGRP staining in the DRG, which suggests that there may have been an increased release of CGRP from peripheral nerves. This is supported by the finding that there was also an increase in circulating CGRP concentrations in animals exposed to vibration. This peptide acts in the periphery to stimulate vasodilation, edema, and pain [227,228]. These findings are consistent with other studies showing a reduction in CGRP-immunostained nerves in finger biopsy samples from workers with HAVS [229]. Based on the results of these studies, measures of changes in responsiveness to transcutaneous electrical stimulation (as measured by the current perception threshold) and circulating CGRP concentrations, might also serve as biomarkers of vibration-induced injury.

### 7.2. Paw Vibration Model

Another animal model developed to study the effects of vibration on vascular physiology was the paw/forelimb model [230]. This model involved restraining an animal in a cone-shaped restrainer but allowing the front right forelimb and paw to reach down to a vibrating platform that was located beneath the animal. The biodynamic responses at various regions on the paw and along the forelimb were measured using a laser vibrometer at frequencies of 32.5, 63, 160, 250 and 500 Hz and 3 accelerations (9.8, 49 and 100 m/s^2^). The resonant frequency was in the range of 125—250 Hz at the paw, using an acceleration of 49 m/s^2^. Based on these findings, animals were exposed to a single 4 h bout of vibration at 125 Hz and 49 m/s^2^, and the dose-responses to phenylephrine and serotonin were measured. The results of these studies demonstrated that exposure to vibration resulted in reduced sensitivity to these vasoconstricting substances in the paw artery. However, vibration also resulted in a rapid increase the oxidative stress in the paw artery. This increase in reactive oxygen species may have acted to reduce the vasoconstriction induced by phenylephrine and serotonin. Restricting vasoconstriction may help maintain blood flow in the short term, however, it may also result in cellular damage, and interfere with normal vasodilating mechanisms in this artery. The results generated in the paw artery are somewhat different than the responses of the tail artery [208] following exposure to a single bout of vibration. These differences may be because the arteries were collected from different locations in the body which affects their sensitivity to various modulators due to differences in the receptors located in different arterial beds. In many rodents, the tail is a thermoregulatory and thermoresponsive structure. The α2C-adrenoreceptor, which responds to changes in temperature, and induces a vasoconstriction in response to vibration or other cellular stressors, is localized in both the tail artery [208] and human fingers [231,232], but not in the artery of the paw [230]. However, the changes in vascular responsiveness in both structures tend to be the result of increases in oxidative damage. Additional studies examining the mechanisms underlying the development of HAVS and markers of altered function or injury may help both early detection and diagnoses of the disorder.

### 7.3. Models of Impact Vibration

A rat tail model was also developed and characterized to examine the effects of impact vibration on vascular and sensorineural function. The system was similar to that used to look at the effects of sinusoidal vibration with the following exception: the Broome style restrainer in which the animal was restrained, was housed in a sound-attenuating chamber to reduce the noise generated during the exposure; the platform the tail was secured to was mounted to a pneumatic riveting hammer (Atlas Copco RRH04P). The vibration characteristics of the platform were measured using a laser vibrometer under different loading and air-pressure conditions. The conditions used for the exposure were 138 kPa with an applied load of 40 N. Using this exposure condition, it was determined that there were resonances around 40, 100 and 300 Hz [233]. This exposure system was used to determine the effects of single, 15 min exposure to impact vibration. A single bout of exposure to impact vibration did not alter the responsiveness of the tail artery to vasoconstriction or dilating factors. Morphological data from peripheral nerves innervating the skin were examined 4 days after the exposure (similar to the protocol used by Zimmerman et al. [234]). The vibration did not affect the number of nerve fibers in the tail, albumin staining (a marker of edema) or mast cell number (a marker of inflammation). However, immunostaining of peripheral nerves using PGP9.5 (a ubiquitin carboxy-terminal hydrolase), which breaks down and recycles proteins in nerve cells and fibers, was increased in peripheral nerves after exposure to impact vibration [235]. Changes in this enzyme have been used as a marker of nerve damage and regeneration [236]. The results of this study suggest peripheral nerves may be more sensitive to impact vibration than peripheral arteries and monitoring peripheral sensory function may serve as an early indicator of impact-induced injury.

### 7.4. Animal Models and Anti-Vibration Materials (VR Gloves)

Many of the anti-vibration materials used in VR gloves have been shown to exhibit a resonant frequency at or close to the resonant frequency of the fingers [35,53,54,237]. To determine if anti-vibration materials reduced the biological effects associated with vibration exposure, the tail model was used to assess the effects of both sinusoidal and impact vibration [166,235]. The results of these studies were consistent with those of human studies; placing air-bladder glove material under the tail to reduce transmission vibration at 125 Hz did not reduce vascular responses to vasoconstricting or vasodilating substances, or increase vascular internal pressure. Additionally, responses to applied pressure were not altered with the use of anti-vibration materials; exposure to vibration resulted in reduced sensitivity to applied pressure both with and without the use of anti-vibration materials [238]. When the biodynamic response of the tail was measured with and without anti-vibration materials (gel and air-bladder), these VR glove materials only seemed to reduce the biodynamic response at frequencies greater than 1000 Hz [166], suggesting that glove materials did not protect tissue from vibration transmission at the most damaging frequencies. New VR gloves are being developed which may provide more protection from the frequencies that are most injurious.

### 7.5. Additional Studies and Data

Exposure to HTV results in changes in blood flow and in sensory perception in the local tissues directly exposed to vibration [132,239]. However, exposure to HTV or segmental vibration has also been shown to induce systemic effects that may be the result of changes in sensitivity to autonomic nervous system signaling [240–243] and possibly various metabolic processes [206,244]. Because results from animal models show segmental vibration exposure is associated with changes in transcript levels of genes associated with an increased risk for developing cancer and heart disease [206,244], additional epidemiological studies looking at the prevalence of these diseases in workers exposed to HTV would help determine if the vibration is a significant risk factor in the development of these diseases. Experimental studies can also contribute by identifying characteristics of vibration that are most likely to contribute to physiological changes associated with the development of these diseases, determine which tissues or biological systems are most sensitive to the effects of vibration, and identify early biomarkers of disease progression so that interventions might be developed and used to prevent disease progression.

There is also a question as to how the age of a worker contributes to the risk of developing HAVS. Although there are studies demonstrating that young workers, with extreme exposures to HTV develop HAVS fairly quickly, in the majority of the studies, most workers with HAVS are older (>50 years of age) and have prolonged exposure to HTV [245–248]. This has led to the following questions: (1) Are there age-related changes in physiology that make it more difficult for older workers to adapt to vibration exposure? and (2) how do other factors that affect a worker’s health contribute to the risk of developing HAVS? Although epidemiological studies have investigated the relationship of some of these factors to the development of HAVS, experimental studies may be able to provide information regarding the contribution of other personal risk factors (e.g., genetic predisposition to, hypertension) on the risk of developing HAVS. There are also more women working with vibrating hand tools [249–251] and because of differences in the responses of peripheral blood vessels to vasomodulating factors, steroid hormone-induced changes in peripheral sensory function and differences in hand size and grip strength, females may respond differently to occupational vibration exposure. Therefore, additional epidemiological and laboratory studies examining the effects of occupational HTV should be done to determine how differences in biology may affect a female’s risk of developing HAVS and if VR devices are as efficient at reducing the transmission of vibration in both males and females.

Because of sex-related differences in the expression of receptors regulating vascular function [231,252], and because changes in estrogen can affect blood flow and sensory perception [253], the effects of vibration can be examined in female rodents to determine how sex-related differences in physiology might affect the responses of the vascular and sensorineural system. Future experiments can also examine how age-related changes in vascular and neural physiology may affect the ability of the body to adapt to and recover from vibration exposure.

## 8. Physiological Measurements of Vibration Health Effects

A number of physiological measures have been used to quantify the effects of occupational HTV exposure on vascular and sensorineural function. They include using measures of blood flow using laser doppler [254–256] and changes in finger systolic blood pressure in response to cold exposure and rewarming [133,199,200,257–267]. Tests of sensorineural function include; nerve conduction [173,203,268–272], vibrotactile sensitivity [95,192,273–279], and tactile sensitivity using von Frey or Semmes Weinstein monofilaments [280,281]. Measures of changes in dexterity have been made using the Perdue Pegboard test and other tests looking at the ability to perform everyday tasks such as buttoning a shirt or zippering a jacket [187,188,282,283]. Vibration-induced changes in grip, pinch or hand/forearm muscle strength have been used measuring electromyography [183,284–289] and grip strength meters [193,290–293]. Many of these studies have produced conflicting results regarding the effects of vibration on these activities. The use of these tests for early detection of vibration-induced dysfunction also has not been examined.

### 8.1. Animal Studies of Vascular Function Using the NIOSH Rat-Tail Model

Thermography has been used in humans to detect vibration-induced vascular function as mentioned above. However, preliminary studies using thermography in our animals demonstrated that tail temperature was reduced in animals exposed to both restraint (in a Broome style restrainer) and tail-vibration in rats, suggesting that although this method is commonly used to detect changes in vascular function in humans it may not be sensitive enough to detect changes in blood flow in the tail model [243,256,294–297]. However, when vibration-induced changes in peripheral vascular function were assessed using laser doppler to measure blood flow, changes were seen in both humans with HAVS and animals [254–256,298–301]. After a single exposure to vibration at 125 Hz (49 m/s^2^), there was a reduction in blood flow in the ventral tail artery that was independent of changes in tail temperature. Blood flow was also measured in response to vibration 5, 10, 15 and 20 d after exposure to vibration. There were no significant changes in overall blood flow on the day’s animals were tested. However, previous studies demonstrated that changes in pulse rate could be detected by using a spectral analysis of the signal to identify the pulse rate [302] from the overall blood flow signal [303]. This analysis demonstrated that on day 15 of vibration exposure (measurements made prior to exposure each day), there was a significant reduction in the amplitude of the arterial pulse [301]. On day 20 of exposure, the amplitude of the pulse was reduced in both restraint controls and vibration exposed. However, the fact that the reduction in the pulse amplitude occurred sooner, and the magnitude of the reduction was more pronounced in animals exposed to vibration, suggests that vibration resulted in an increase in stiffness of the artery, which could be due to a thickening of the smooth muscle wall [204,206,252,304]. These findings are consistent with those seen in humans that have been exposed to vibration [255,256,299] and with changes in vascular morphology indicative of vascular dysfunction [204,206].

### 8.2. Animal Studies of Sensorineural Function Using the NIOSH Rat-Tail Model

Studies were performed to explore non-invasive tests that could be used to assess sensorineural function in the rat tail model that could also be used in humans. Previous studies had demonstrated the current perception threshold (CPT) might be a useful measure for detecting early changes in peripheral sensory nerve function with exposure to vibration [226,268,305]. Workers with HAVS showed changes in CPT measures as compared to controls. However, their scores did not fall into the range of people with neuropathies according to established standards [268]. To determine if CPT test scores were associated with markers of peripheral nerve damage, this test was used to correlate changes in nerve function with changes in morphology in rats that had been exposed to vibration. The CPT test uses transcutaneous electrical stimulation at 3 frequencies, 2000, 250 and 5 Hz, to test the functioning of large-myelinated Aβ-fibers which carry information about mechanical stimuli, small-myelinated Aδ-fibers, which carry information about light touch, and unmyelinated C-fibers, which carry information about pain, respectively [268,306,307]. A stimulating electrode and a dispersing electrode are attached to the animals’ tail (or the portion of the human body to be tested) and an electrical current at one of the 3 frequencies listed above is applied. The amplitude of the current is gradually increased until the animal moves its tail or the human says that they feel the stimulus. The amplitude that elicits a response is referred to as the CPT for that frequency. This test bypasses sensory receptors and directly tests nerve function [268,306,307]. Using this method, the lab has demonstrated that the CPT is not altered by the temperature of the tail [306,308], and that changes in the CPT, particularly when using the 2000 Hz stimulus, are associated with markers of peripheral nerve dysfunction and injury [201,216,225,309]. This is similar to what has been seen in humans and is consistent with the idea that the CPT test is a non-invasive procedure that can be used to detect early changes in peripheral nerve function associated with occupational exposure to HTV [226,268,305].

Tests of touch or applied pressure have also been used to detect changes in sensorineural function after exposure to occupational HTV in humans diagnosed with HAVS [280,281] and in animals exposed to vibration [201,217,238,310]. In humans, sensitivity to tactile stimulation or applied pressure is usually measured with von Frey or Semmes Weinstein monofilaments [223,280,281]. Both these tests involve placing a monofilament of a specific tensile strength on the body area to be tested. Pressure using the filament is applied until the subject says they feel the pressure or until the filament bends. This stimulus is applied first in an order where the tensile strength of the filament is increased until the subject responds, and then in descending order, where a filament that induces a response is used first, and the tensile strength is gradually decreased until the subject does not respond. Studies using monofilament have shown that workers with HAVS display a reduced sensitivity to touch or applied pressure [223,280,281]. However, studies using monofilaments to examine changes in tactile sensitivity in the NIOSH rat tail model did not find effects of vibration exposure on tactile sensitivity, even when there was evidence of nerve damage and changes in the CPT [201]. This may be due to the fact that animals quickly adapted to the test in these experiments, and the lightest touch induced a response after the first trial. However, because human studies suggested that tactile sensitivity may be an early indicator of vibration-induced sensorineural dysfunction in humans, an aesthesiometer was used to determine responses to applied pressure in control and vibration exposed animals. To perform this test, forceps connected to a force meter are used to precisely measure the applied pressure. During the test, pressure is applied to the tail, and once the animal flicks their tail the pressure source is removed, and the applied force is recorded on the meter. The meter can be set to limit the amount of force that can be applied to prevent injury. Studies performed using this meter found that initial exposures to vibration resulted in increases in sensitivity to applied pressure, but with longer exposures, sensitivity to applied pressure may decrease [238,310], as it does in humans. The advantage of using the force meter is that a precise measurement of the amount of pressure needed to induce a response is recorded, and that the test could be adapted for use in people. Therefore, although additional studies need to be performed, it is possible that testing tactile sensitivity or sensitivity to applied pressure might be a good diagnostic tool to detect the early effects of vibration-induced sensorineural dysfunction.

### 8.3. Automated Nail Blanching Test

A nail blanching test could help detect VWF. A prototype of an automated nail blanching test device has been developed by NIOSH researchers [311]. It may be directly used or combined with the other physiological tests (e.g., a cold challenge test) to help detect VWF. Further development and experimental studies are required to test this hypothesis.

### 8.4. Improved Thermal Perception Threshold Test

The measurement of increased thermal perception threshold (TPT) or decreased sensitivity of the mechanoreceptors in persons is considered an alternative approach for the objective early detection of vibration-induced sensorineural disorders [297,312–314]. An aesthesiometer is usually used to measure the TPT. To improve the test method, NIOSH researchers proposed an automatic method for controlling the finger force applied on the aesthesiometer and tested the following hypotheses [315]: (i) the method for controlling finger force during the test would affect the magnitudes of the TPTs; and (ii) the variation in finger force levels would affect the magnitudes of the TPTs. This study concluded that it was not necessary to tightly control the finger force but it was better to use the middle range force (2 to 8 N) for the test. The automatic force control device can help achieve more sensitive TPT measurements.

## 9. Summary and Major Areas for Future Research

This review provided a summary and elaboration of the major studies on hand-transmitted vibration exposure and health effects conducted by the researchers in the Health Effects Laboratory Division of NIOSH. The major areas identified for future research include the following aspects:

- The fatigue-failure theory applied to vibration exposure and health effects has not been well established. While the combination of further experimental and FE modeling studies can provide reliable quantifications of various vibration dose measures, further biological studies are needed to test each of them. Most of the published biological studies examined the association between vibration exposure factors (vibration acceleration magnitude, frequency, and duration) and vibration biological effects, which includes biodynamic and biological processes. There is still a lack of information on the specific role of each process in determining the biological effects. It remains unknown what the quantitative relationship between a detailed biodynamic response (stress, strain, or VPAD) and a biological effect in the body or exposed appendage, which is the critical part of the vibration fatigue-failure theory. Biological models should be designed such that the biodynamic responses can be conveniently measured and controlled and the biological studies can focus on the second process: from biodynamic responses to the biological effects. This may require synergized efforts by biodynamic and biological researchers.
- While the overall psychophysical responses such as the vibration sensation, discomfort, and pain of the entire hand–arm system have been investigated and the results have been used as a basis to determine the standard frequency weighting of the hand-transmitted vibration exposure, few studies have examined the relationship between the location-specific vibration biodynamics and psychophysical responses [99]. Further studies in this aspect may help determine the location-specific frequency weightings of the HTV exposure.
- Applied hand forces, hand–arm postures, and vibration exposure direction may significantly affect biodynamic and physiological responses but they have not been considered in the standard method for the HTV risk assessment. Further studies are required to develop more effective devices for their measurement and to determine their specific weightings in the formulation of the HTV exposure dose.
- It is highly desired to have a reliable and convenient device to measure and monitor the vibration exposure of workers at workplaces. Further studies are required to apply advanced technologies to improve HTV dosimeters and to develop effective HTV exposure direct-reading devices.
- The current diagnosis of the hand–arm vibration syndrome depends on the use of a combined subjective survey and some measurement technologies. Some misdiagnosis may happen. Further studies are required to develop more reliable objective methods/devices for the diagnosis of HAVS. A reliable dose–effect relationship can be established only when the vibration exposure dose that truly reflects the exposure factors can be formulated and the vibration health effects can be reliably quantified.
- It remains a research challenge to develop more effective VR tools and devices without decreasing productivity and/or causing other safety concerns. Further development and application of mechanical arms and exoskeletons may help design more effective VR tools and devices. Further studies are also required to evaluate these new technologies and to minimize their adverse effects. The development and application of other new intervention methods and technologies that can decrease the required hand forces, avoid awkward hand and arm postures, and increase safe work practices (e.g., keeping hand warm and dry, and reducing noise exposure) may also help control HAVS.

One of the objectives of this review was to clarify some of the information used in improving international and national standards, guidelines, and educational materials related to hand-transmitted vibration exposure [4,23,33,38,41–43,121,123,295,316–318]. It is our hope that this review will stimulate interest among researchers in addressing the gaps in the hand–arm vibration knowledge base.

## Data Availability

No new data were created and/or reported in this review paper.
